# High-Resolution Digital Phenotypes From Consumer Wearables and Their Applications in Machine Learning of Cardiometabolic Risk Markers: Cohort Study

**DOI:** 10.2196/34669

**Published:** 2022-07-29

**Authors:** Weizhuang Zhou, Yu En Chan, Chuan Sheng Foo, Jingxian Zhang, Jing Xian Teo, Sonia Davila, Weiting Huang, Jonathan Yap, Stuart Cook, Patrick Tan, Calvin Woon-Loong Chin, Khung Keong Yeo, Weng Khong Lim, Pavitra Krishnaswamy

**Affiliations:** 1 Institute for Infocomm Research Agency for Science Technology and Research (A*STAR) Singapore Singapore; 2 SingHealth Duke-NUS Institute of Precision Medicine Singapore Singapore; 3 SingHealth Duke-NUS Genomic Medicine Centre Singapore Singapore; 4 Cardiovascular and Metabolic Disorders Program, Duke-NUS Medical School Singapore Singapore; 5 Department of Cardiology National Heart Centre Singapore Singapore Singapore; 6 Duke-NUS Medical School Singapore Singapore; 7 Cancer and Stem Biology Program, Duke-NUS Medical School Singapore Singapore; 8 Cancer Science Institute of Singapore National University of Singapore Singapore Singapore; 9 Genome Institute of Singapore Agency for Science Technology and Research (A*STAR) Singapore Singapore

**Keywords:** wearable device, heart rate, cardiometabolic disease, risk prediction, digital phenotypes, polygenic risk scores, time series analysis, machine learning, free-living

## Abstract

**Background:**

Consumer-grade wearable devices enable detailed recordings of heart rate and step counts in free-living conditions. Recent studies have shown that summary statistics from these wearable recordings have potential uses for longitudinal monitoring of health and disease states. However, the relationship between higher resolution physiological dynamics from wearables and known markers of health and disease remains largely uncharacterized.

**Objective:**

We aimed to derive high-resolution digital phenotypes from observational wearable recordings and to examine their associations with modifiable and inherent markers of cardiometabolic disease risk.

**Methods:**

We introduced a principled framework to extract interpretable high-resolution phenotypes from wearable data recorded in free-living conditions. The proposed framework standardizes the handling of data irregularities; encodes contextual information regarding the underlying physiological state at any given time; and generates a set of 66 minimally redundant features across active, sedentary, and sleep states. We applied our approach to a multimodal data set, from the SingHEART study (NCT02791152), which comprises heart rate and step count time series from wearables, clinical screening profiles, and whole genome sequences from 692 healthy volunteers. We used machine learning to model nonlinear relationships between the high-resolution phenotypes on the one hand and clinical or genomic risk markers for blood pressure, lipid, weight and sugar abnormalities on the other. For each risk type, we performed model comparisons based on Brier scores to assess the predictive value of high-resolution features over and beyond typical baselines. We also qualitatively characterized the wearable phenotypes for participants who had actualized clinical events.

**Results:**

We found that the high-resolution features have higher predictive value than typical baselines for clinical markers of cardiometabolic disease risk: the best models based on high-resolution features had 17.9% and 7.36% improvement in Brier score over baselines based on age and gender and resting heart rate, respectively (*P*<.001 in each case). Furthermore, heart rate dynamics from different activity states contain distinct information (maximum absolute correlation coefficient of 0.15). Heart rate dynamics in sedentary states are most predictive of lipid abnormalities and obesity, whereas patterns in active states are most predictive of blood pressure abnormalities (*P*<.001). Moreover, in comparison with standard measures, higher resolution patterns in wearable heart rate recordings are better able to represent subtle physiological dynamics related to genomic risk for cardiometabolic disease (improvement of 11.9%-22.0% in Brier scores; *P*<.001). Finally, illustrative case studies reveal connections between these high-resolution phenotypes and actualized clinical events, even for borderline profiles lacking apparent cardiometabolic risk markers.

**Conclusions:**

High-resolution digital phenotypes recorded by consumer wearables in free-living states have the potential to enhance the prediction of cardiometabolic disease risk and could enable more proactive and personalized health management.

## Introduction

### Background

The adoption of consumer-grade wearable activity trackers into routine use has been increasing rapidly in recent years, with approximately 1 in 5 adults in the United States reported to regularly use wrist-worn smartwatches and fitness trackers in 2019 [1]. This phenomenon has generated an unprecedented scale of consumer health data and led to many studies on the wider health uses of such data. These studies are increasingly generating evidence to reveal relationships between recordings from wearable activity trackers and the risk for conditions ranging from mental health and infectious diseases [2,3] to cardiovascular and metabolic (collectively referred to as *cardiometabolic*) diseases [4-7]. Among these, owing to the apparent links between activity levels and cardiometabolic health, the evidence for broader health uses of wearables is most established in the cardiometabolic domain [4,8-11].

Previous studies in the cardiometabolic domain have focused on the utility of wearable-derived summary statistics, and fall into 1 of 2 categories. First, electrocardiogram signals from wearables have been studied in relation to the development of cardiometabolic conditions, such as atrial fibrillation [12-14], hyperkalemia [15,16], and heart failure [17-19]. As many of these conditions are amenable to early intervention via dietary changes or increased physical activity, there is also an interest in using wearables to promote self-awareness and regulation [20] and to enhance screening [11]. Second, wearable-derived measures, such as circadian measures, sleep patterns and quality [11,21], step counts [4], wearable-derived resting heart rate [4,8,10,21,22] and heart rate variability [23-27] have been found to correlate with outcomes in cardiometabolic disease. As such, there is increasing recognition in the clinical community to incorporate wearable-derived measures into practical cardiometabolic disease management [6,28].

### Objectives

Rapid and ongoing developments in consumer wearable technology are enabling ever-richer measurements with finer temporal resolution for heart rate, activity, and sleep dynamics in free-living states [6,29,30]. Principled analyses of such data streams could generate new insights beyond summary statistical measures for cardiometabolic health and disease management. However, the analysis of time series data recorded in free-living states is challenging, as these data tend to exhibit real-world noise and fluctuations and typically lack important physical and physiological contexts. A few recent studies have used black-box deep neural networks to relate high-resolution heart rate and step count time series recorded using wearables to the risk of developing atrial fibrillation, sleep apnea, and hypertension [31,32]. As their primary goal focused on risk target classification, the nature of the intermediate predictive time series features and their connection with known clinical and biological markers of cardiometabolic disease remains unresolved.

In this study, we aimed to derive high-resolution digital phenotypes from consumer wearable heart rate recordings and to examine their associations with diverse risk markers for cardiometabolic disease. Specifically, we sought to develop a time series feature extraction approach, contextualized by activity state, to meaningfully represent heart rate dynamics recorded by consumer wearables in free-living conditions. We then applied our approach to multidimensional data from normal volunteers in the SingHEART study [33] to assess the extent to which the derived high-resolution wearable features could predict expressed clinical risk markers for cardiometabolic disease. Furthermore, we assessed whether these high-resolution features also represent more subtle physiological changes associated with an inherent genetic predisposition to cardiometabolic disease. Finally, we qualitatively characterized these wearable phenotypes in volunteers who had actualized clinical events to assess connections beyond risk markers to manifest cardiometabolic diseases.

## Methods

### Data

We sourced data from the SingHEART study (NCT02791152) as of October 8, 2019. Enrollment targeted healthy volunteers who provided written informed consent to use the data (including electronic health records) for research. Participants were required to fulfill the inclusion criteria presented in Textbox 1.

**Inclusion criteria.**

**Inclusion criteria**

1. 21-69 years of age
2. No personal medical history of prior cardiovascular disease (myocardial infarction, coronary artery disease, peripheral arterial disease, stroke), cancer, autoimmune or genetic disease, endocrine disease, diabetes mellitus, psychiatric illness, asthma, chronic lung disease, or chronic infectious disease
3. No family medical history of cardiomyopathies

At enrollment, each participant was profiled using a range of health assessment modalities. The resulting data set included (1) heart rate and step count time series recordings over 3 to 5 days from consumer wearable devices (Fitbit Charge HR), together with the associated sleep logs generated by Fitbit, (2) self-reported answers to a lifestyle and quality-of-life questionnaire [4], (3) genotypic data from whole genome sequencing using the Illumina HiSeq X platform, and (4) laboratory measurements for 9 clinically relevant markers (systolic and diastolic blood pressure; blood levels of triglycerides, total cholesterol, high-density lipoprotein, and low-density lipoprotein; fasting blood glucose level; waist circumference and BMI). As of October 8, 2019, the full study cohort contained 1101 participants, of whom 692 (62.8%) participants had wearable recordings. We focused on this subset of participants for subsequent analysis: a detailed breakdown of the data is provided in Table 1.

Furthermore, we also tracked each participant for the occurrence of any actual clinical event. We extracted all clinical codes (based on the International Classification of Diseases, 10th Revision) pertaining to any acute care use events in the regional health system associated with the National Heart Centre Singapore until January 2021 to characterize the links among data features, risk markers, and actual clinical events.

**Table 1:** Summary of demographic, clinical, and consumer wearable data for participants with wearable recordings (N=692) in the SingHEART study cohort.

	Female (n=370, 53.5%)			Male (n=322, 46.5%)	
	Value, mean (SD)	Participants, n^a^ (%)	Value, mean (SD)		Participants, n^a^ (%)
Age (years)	45.47 (11.71)	0 (0)	44.46 (13.29)		0 (0)
BMI (kg/m^2^)	22.87 (3.94)	0 (0)	24.33 (3.39)		0 (0)
WC^b^ (cm)	78.91 (10.98)	0 (0)	86.96 (9.86)		0 (0)
SBP^c^ (mm Hg)	122.51 (17.74)	0 (0)	132.20 (14.96)		0 (0)
DBP^d^ (mm Hg)	73.38 (12.80)	0 (0)	82.18 (10.97)		1 (0.3)
Wearable-derived resting heart rate (bpm; Fitbit)	70.66 (6.55)	0 (0)	69.37 (6.59)		0 (0)
ECG_HR^e^ (bpm)	64.46 (9.17)	10 (2.7)	63.67 (9.87)		12 (3.7)
Total cholesterol (mmol/L)	5.34 (0.94)	6 (1.6)	5.33 (0.97)		5 (1.6)
LDL^f^ (mmol/L)	3.32 (0.81)	7 (1.9)	3.40 (0.89)		6 (1.9)
HDL^g^ (mmol/L)	1.59 (0.32)	6 (1.6)	1.36 (0.30)		5 (1.6)
TGs^h^ (mmol/L)	0.99 (0.51)	6 (1.6)	1.30 (0.76)		5 (1.6)
Glucose (mmol/L)	5.17 (0.49)	8 (2.2)	5.36 (0.71)		5 (1.6)
Average daily step count^i^	10,349.81 (4180.35)	30 (8.1)	10,972.86 (3919.10)		20 (6.2)
Average daily sedentary minutes	633.45 (96.48)	102 (27.6)	656.49 (95.58)		88 (27.3)
Average daily sleep minutes	395.92 (61.18)	102 (27.6)	374.49 (65.15)		88 (27.3)

^a^Refers to number of participants with missing or incomplete values for the respective fields.

^b^WC: waist circumference.

^c^SBP: systolic blood pressure.

^d^DBP: diastolic blood pressure.

^e^ECG_HR: electrocardiogram heart rate.

^f^LDL: low-density lipoprotein.

^g^HDL: high-density lipoprotein.

^h^TG: triglyceride.

^i^The average daily step count was derived by taking the sum of steps for each day and then averaging over days. Only days with ≥20 hours of valid data were considered.

### Ethics Approval

The SingHEART study (NCT02791152) was established at the National Heart Centre Singapore, a tertiary specialty hospital in Singapore, and was approved by the SingHealth Centralized Institutional Review Board (ref: 2015/2601 and 2018/3081) [33,34].

### A Set of 22 Canonical Time Series Characteristics

Given a time series segment, it is possible to define a set of high-resolution features using approaches such as the highly comparative time series analysis [35,36] and time series feature extraction on the basis of scalable hypothesis [37,38]. However, such approaches can generate many redundant features, and the process of selecting a concise but effective representation is often not straightforward. A recent study [39] introduced a minimally redundant and interpretable set of 22 features, termed as Canonical Time-series Characteristics 22 (Catch22) features, which have high predictive value across 93 diverse time series classification data sets. As this Catch22 feature set was designed to reduce interfeature redundancy, it provides a compendious representation of the different dynamic properties of the time series.

The Catch22 features fall into seven main categories, namely (1) distribution, (2) extreme events, (3) symbolic, (4) linear autocorrelation and periodicity, (5) nonlinear autocorrelation, (6) successive differences, and (7) fluctuation analysis. The distribution-based features represent summary statistics of the distribution of the measured values in the series (while ignoring the chronological order of these values). The extreme event features represent intervals between successive outlier events in the time series. The symbolic features represent statistics summarizing the outputs of symbolic transformations of the actual time series values. The linear autocorrelation and periodicity features comprise summary statistics on inherent periodicities in the time series. The nonlinear autocorrelation features involve summary statistics on periodicities based on nonlinear transformations of the time series. The successive difference features represent statistics based on the time series of the incremental differences. Finally, the fluctuation analysis features quantify the statistical self-affinity of the time series. Detailed descriptions of each of the 22 features are provided in Table S1 in Multimedia Appendix 1.

### Extraction of Features From Wearable Time Series Recordings

We now describe the steps to derive resting heart rate, summary statistics on activity and sleep patterns, and high-resolution features from the wearable heart rate and step count time series recordings. As all these physiological features are derived from the same recordings, they are internally consistent and can be meaningfully used for downstream comparative analyses.

#### Computation of RestingHR

We used wearable heart rate time series recordings to derive resting heart rate [4]. Specifically, we defined *wearable-derived resting heart rate* as the average of heart rate values across all time points that had a valid heart rate record and a step count of ≤100. We note that there are similarities between the wearable-derived resting heart rate and the clinical gold standard electrocardiogram-derived heart rate [4,40].

#### Annotation of Wearable Time Series Recordings

**Figure 1:**
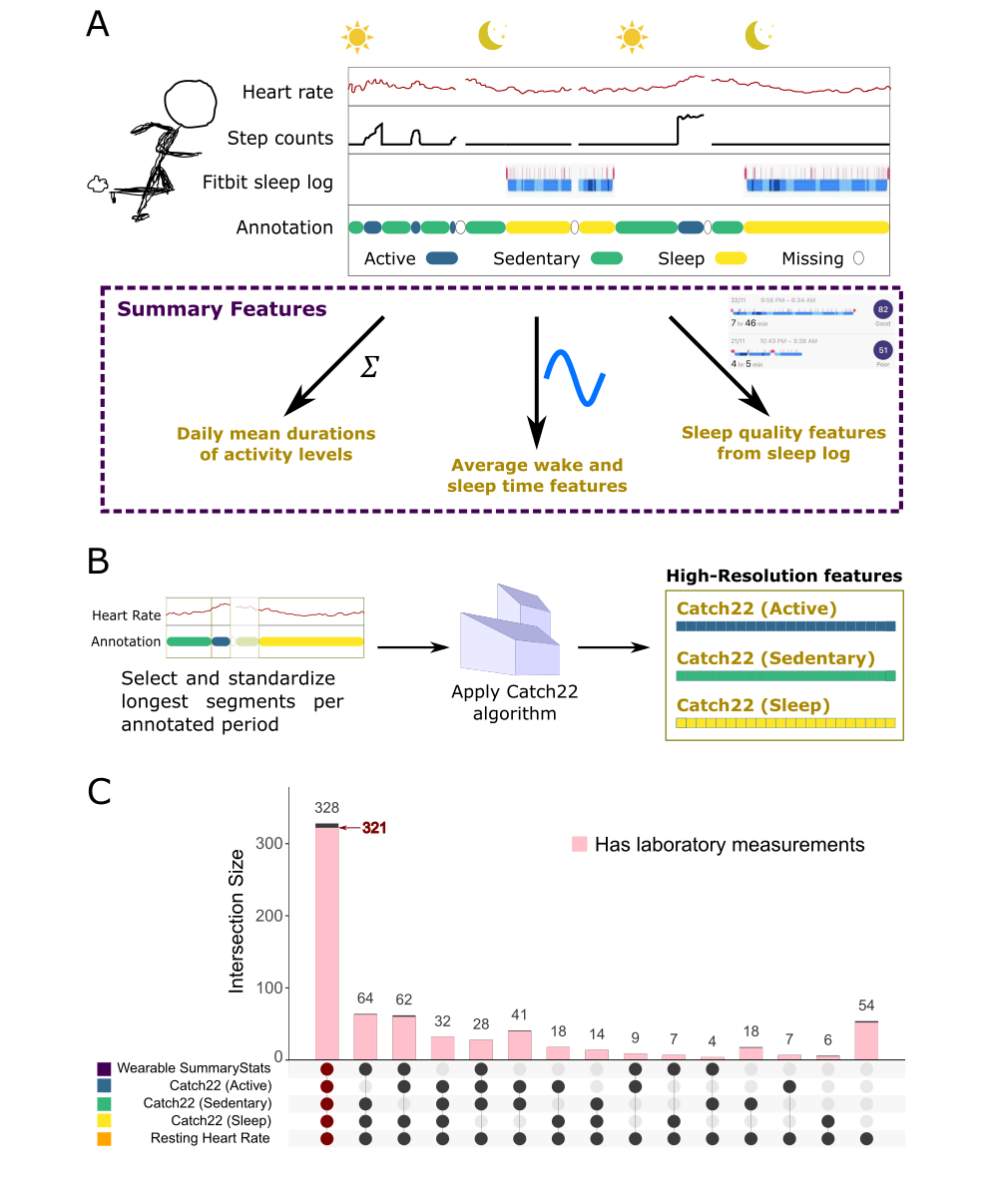
Wearable data processing pipeline. (A) Construction of low-resolution features based on summary statistics. (B) Construction of high-resolution features based on the Canonical Time-series Characteristics 22 (Catch22) algorithm. (C) UpSet plot of the 692 participants with features from the various categories. Only nonempty set intersections are presented. Intersection size indicates the number of participants found within the intersections of given sets. Of the largest intersection with 328 participants, 321 also had laboratory measurement recordings.

We extracted the wearable time series recordings for each participant and used only days with at least 20 hours of step count and heart rate data as per Lim et al [4]. Heart rate recordings were available either at regular 1-minute intervals or as irregular bursts of recordings over 5-, 10-, or 15-second intervals. Step count recordings were sampled at either 15-minute or 1-minute intervals. We resampled all heart rate and step count consumer wearable records to 1-minute intervals and then annotated the time series to reflect data availability and physical activity states (Figure 1A). We assigned a *null* value for heart rate at time points where it was missing. Then, we annotated time points with available data for both heart rate and step count as “sleep,” “active,” or “sedentary.” Specifically, we applied the *sleep* annotation to all time points captured by the Fitbit sleep log, the *sedentary* annotation to any time points with 0 step count value, and denoted the remaining time points as *active*. On average, the participants in our study had 3.72 days of valid heart rate data, and the average missing heart rate periods in a day were 94.9 (SD 85.8) minutes. The median lengths of the longest uninterrupted time series for the *active*, *sedentary*, and *sleep* periods were 31, 105, and 465 minutes, respectively.

Subsequently, we processed the heart rate and step count time series recordings from the consumer wearable devices to yield a range of summary and high-resolution features, as detailed in subsections *Derivation of Summary Features From Wearable Time Series Recordings* and *Derivation of High-Resolution Features From Wearable Time Series Recordings*.

#### Derivation of Summary Features From Wearable Time Series Recordings

We used a 3-step procedure to derive a range of wearable summary statistics (Figure 1A). First, we used our physical activity annotations to compute mean daily durations for the different activity states. Second, we used device logs to obtain statistics relating to sleep-wake patterns. Third, we converted the wake and sleep times into a 24-hour format and averaged the resulting values over all days where a given participant had wearable data recordings. To account for the cyclical nature of sleep or wake patterns, we transformed the average wake and sleep times using sinusoidal functions. Overall, this process yielded 10 summary features for each participant. All summary statistics are listed in Table S2 in Multimedia Appendix 1.

#### Derivation of High-Resolution Features From Wearable Time Series Recordings

We further developed a data processing pipeline to extract high-resolution time series features from heart rate recordings of the wearable device (Figure 1B). As heart rate and step count patterns under different physical activity states could provide distinct insights into cardiovascular health, we sought to derive time series features that encode contextual information about the physical activity state. Specifically, we processed heart rate time series recordings for each of the 3 physical activity states (sleep, sedentary, and active) separately, as follows.

For each participant, we chose the longest uninterrupted period of the heart rate time series recordings for each physical activity state. As the data exhibit significant variability in the lengths of these periods across participants, we defined prespecified lengths to extract standardized sleep, sedentary, and active segments. Specifically, we extracted the first 20 minutes for active segments, the first 1 hour for sedentary segments, and the first 5 hours for sleep segments. If the recordings available for a participant did not fulfill the prespecified length criteria, even with the longest segment for a given activity state, we did not consider that particular activity state for high-resolution analyses. This process yielded up to 3 heart rate time series segments for each participant.

For each available heart rate time series segment, we applied the Catch22 methodology [39] to obtain 22 high-resolution features. Collectively, our pipeline resulted in up to 3 sets of 22 high-resolution features per participant, namely Catch22 (Sleep), Catch22 (Active), and Catch22 (Sedentary).

As our study did not prescribe controlled experimental settings for the wearable recordings, the resulting time series segments often exhibit significant noise and irregularities. Hence, we considered the reliability of our feature representation approach in these real-world settings. In particular, we assessed stability and sensitivity of the Catch22 features to the length specifications across activity states (Section SI-1, Multimedia Appendix 1). The results suggest that the features are relatively robust within the intervals considered and provide confidence for the downstream use of these high-resolution features.

#### Overlap Among Features Derived From Wearable Time Series Recordings

Figure 1C illustrates the overlaps among participants with the different wearable-derived features using UpSet plots [41,42]. For example, 41 individuals had features for active and sedentary segments but did not have sleep segments or summary statistics (owing to a lack of sufficiently long continuous sleep recordings). We note that all the different types of wearable features are available for 328 participants, of which 321 (97.9%) also had laboratory measurements. We considered this set of 321 participants for ensuing visualization, risk modeling, and analysis.

### Visualization of High-Resolution Heart Rate Features From Wearables

**Figure 2:**
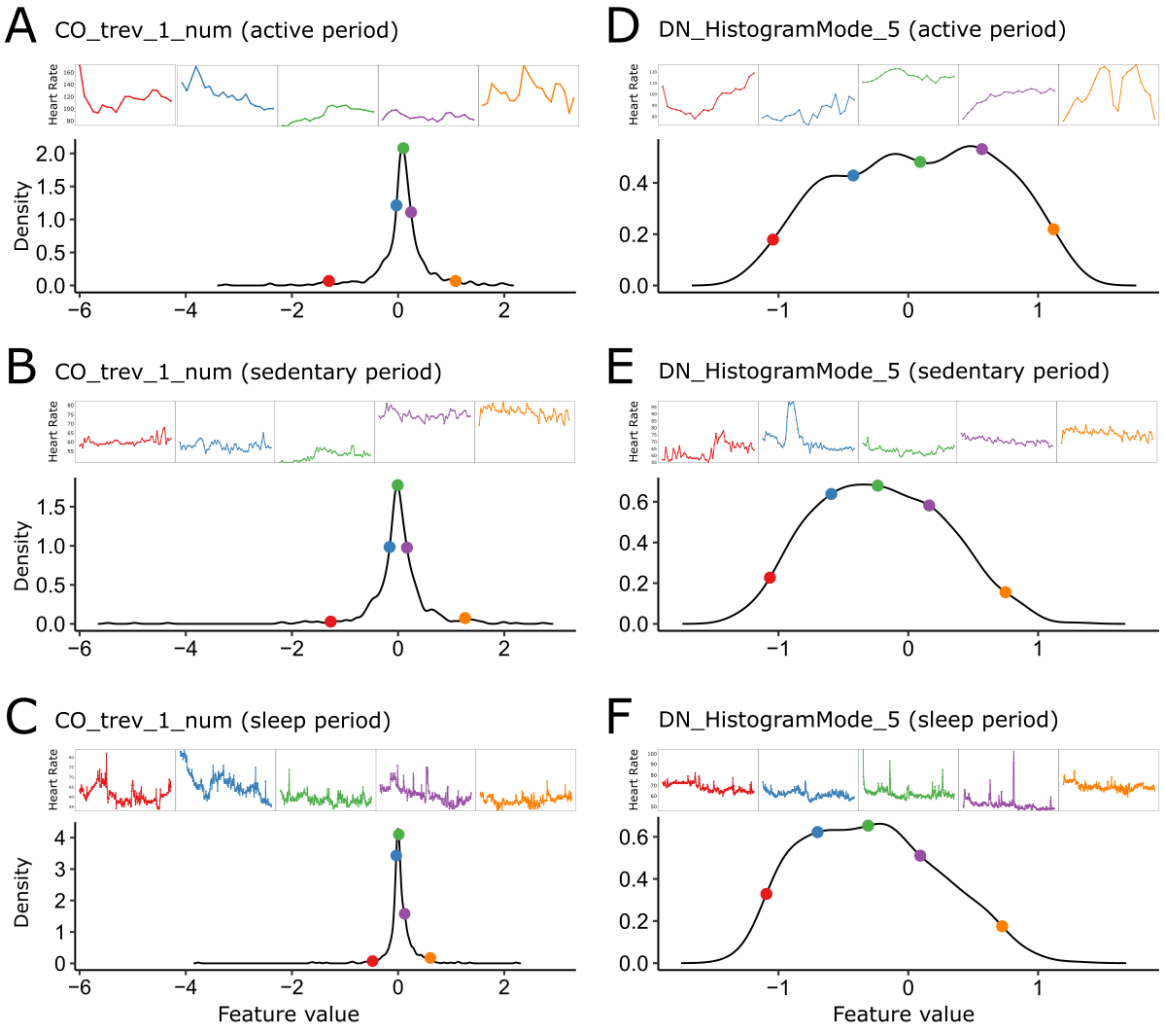
Illustration of wearable-derived high-resolution heart rate features. The distributions of 6 high-resolution features from the 321 participants, based on 2 Canonical Time-series Characteristics 22 features obtained from time series recordings in each of the 3 activity levels. The selected participants are at the 2.5th, 25th, 50th, 75th and 97.5th percentiles of each distribution, and the time series for the participant is plotted in the corresponding color. (A-C) CO_trev1_num is the time-reversibility statistic; higher values tend to correspond to “spikier” or irregular time series. (D-F) DN_HistogramMode_5 takes a time series and groups the z-scored values into 5 linearly spaced bins and reports the mode of the bins.

We examined how high-resolution wearable-derived heart rate features from sleep, active, and sedentary segments were distributed across study participants. Figure 2 illustrates the empirical distributions of exemplar features drawn from segments corresponding to each of the 3 physical activity states. To examine the variability across participants, we also visualized representative time series at the 2.5th, 25th, 50th, 75th, and 97.5th percentile of the density.

The first example comprises a nonlinear autocorrelation feature (CO_trev1_num*,* quantifying the time-reversibility statistic <(x_t+1_-x_t_)^3^>_t_) that relates to the degree of spikiness or regularity in the wearable-based heart rate time series (Figures 2A-2C). The second example comprises a distribution feature (DN_HistogramMode_5*,* corresponding to the mode of the z-transformed values) that quantifies the degree of nonnormality of the time series values by representing the difference between the most probable values (mode) and the mean of the series (Figures 2D-2F).

### Characterization of Predictive Value of Wearable-Derived Features for Clinical Targets

#### Overview

The overall approach used to characterize the predictive value of different wearable-derived features with respect to a variety of clinical risk markers is as follows. Specifically, we considered model types based on 6 different feature sets (Table 2). We then defined 4 target clinical risk markers based on whether the 9 laboratory measurements exceeded the thresholds in Table 3: (1) abnormal blood pressure readings (*bp_abnormal*); (2) abnormal lipid levels (*lipids_abnormal*) for at least one of III to VI; (3) obese (*obesity*) for either VIII or IX; and (4) an omnibus category for lipid, blood sugar, obesity and sugar abnormalities (*anyRISKoutof9*) for any of I to IX.

All 321 participants who had a complete set of wearable-derived features also had complete data for the 9 laboratory measurements. We considered this set of 321 participants as our training set to model the clinical risk targets. Of these 321 participants, 149 (46.4%) were not positive for any of the 4 risk markers, whereas 172 (53.5%) were positive for at least one risk marker (Section SI-2, Multimedia Appendix 1). We noted that a given participant can be positive for >1 of the 4 labels, but most participants exhibiting positive risk markers were exclusively labeled by a single risk marker. Of the 172 positive participants, 119 (69.2%) were positive for 1 clinical risk marker, 40 (23.3%) were positive for 2 risk markers, and only 14 (8.1%) were positive for 3 or more risk markers.

**Table 2:** Description of the different model types.

Model name	Features included	Features, n
Baseline [4]	Age+gender	2
RestingHR	Baseline features+wearable-derived resting heart rate	3
SummaryStats	Baseline features+wearable summary stats	12
HighRes.ActiveSeg	Baseline features+Catch22^a^ (active)	24
HighRes.SedenSeg	Baseline features+Catch22 (sedentary)	24
HighRes.SleepSeg	Baseline features+Catch22 (sleep)	24

^a^Catch22: Canonical Time-series Characteristics 22.

**Table 3:** Laboratory measurements and corresponding thresholds.

Laboratory measurement		Threshold to be considered at risk
I. Systolic blood pressure (mm Hg)		>140
II. Diastolic blood pressure (mm Hg)		>90
III. Triglycerides (mmol/L)		>2.3
IV. Total cholesterol (mmol/L)		>6.2
V. HDL^a^ (mmol/L)		<1
VI. LDL^b^ (mmol/L)		>4.1
VII. Fasting blood glucose level (mmol/L)		>6
**VIII. Waist circumference (cm)**		
	Male	>100
	Female	>90
IX. BMI (kg/m^2^)		>27.5

^a^HDL: high-density lipoprotein.

^b^LDL: low-density lipoprotein.

We used machine learning to model the complex nonlinear relationships between a given feature set and the target pairing using 2 separate approaches. First, for any given target, we analyzed the predictive value of different feature sets (Table 1) using a model comparison approach. Specifically, we consider the degree to which the wearable-derived features (resting heart rate, wearable summary statistics, and different high-resolution wearable features) augment the predictive value of the baseline demographic feature set and also compared the performance of the high-resolution wearable features with that of the lower-resolution features. For an appropriate comparison of value addition over the baseline features, all feature sets based on wearable data also included the corresponding baseline features. Second, for each prediction target, we also compared the importance of the individual feature variables. To have a common basis for these variable importance calculations, we developed a unified model with all features included, and used this model to compare variable importance for the different features.

#### Prediction Model and Variable Importance

We trained machine learning models to estimate the probability that a participant exhibits clinical risk markers for common cardiometabolic disease abnormalities. Specifically, we used random forest classifiers [43] to model the 4 targets of interest, as they are general purpose, nonlinear classifiers that perform well in diverse settings. We trained the random forest models in R using the *randomForest* package [44]. To handle the imbalanced nature of the prediction tasks at hand, we set the number of minority class samples chosen for each tree at 80% of the total minority class size. We then down-sampled the majority class to match the number of minority class samples used [45]. This was implemented using the *strata* and *sampsize* parameters. For each of the 4 prediction targets, we constructed 200 such random forests with different starting random seeds, and for each random forest trained, we recorded the out-of-bag (OOB) prediction errors.

For random forests, variable importance can be quantified using the mean decrease in accuracy (MDA) over all OOB cross-validated predictions. To obtain statistically robust estimates of variable importance, for a given prediction target, we averaged the MDA for each feature across the 200 random forests and then ranked the features by their average MDA to obtain the top 10 important features. To visualize the variable importance results, we considered the union of the top 10 ranking features for the 4 cardiometabolic disease risk targets.

#### Model Performance Metric and Assessment

As the risk prediction task is inherently probabilistic, a suitable metric for model performance assessment would emphasize the calibration of the model predictions (ie, the prediction probabilities of true positives and true negatives are close to 1 and 0, respectively). Therefore, we evaluated the accuracy of probabilistic predictions using the Brier score [46]:

BrierScore(*M*) = [∑_i=1_*^N^* (*p*_i_ - *o*_i_)^2^] / *N* **(1)**

where *M* is the wearable-based model under consideration, *p_i_* is the prediction probability of observing target *i* using the model under evaluation, *o_i_* is the actual observed target or label (binary:0/1), and *N* is the total number of participants included in the modeling. The Brier score ranges from 0 to 1 and is lower for models with better calibrated predictions.

We used OOB estimates [43,47] to evaluate the scores, as there were insufficient data for an independent held-out test set. In total, the above process yielded 200 Brier scores for each pairing of the prediction target and wearable-derived feature set (model) type.

For each target, we also compared the performance of the various model types in relation to each other. Specifically, for each pair of model types, we performed a 2-tailed Welch *t* test on the null hypothesis that the true difference in Brier scores was 0. For each target, we corrected for multiple hypothesis testing by controlling the false discovery rate [48].

### Characterization of Associations Between Wearable-Derived Features and Genomic Risk Markers

To better understand the nature of wearable-derived time series features, we investigated their associations with genomic risk markers for cardiometabolic disease. As probing these associations requires handling diverse multidimensional data types with potentially complex nonlinear relationships, we used a machine learning framework (similar to the one described earlier) to model these relationships. We then used model performance measures to infer the degree of information overlap between wearable features and genomic risk targets. As genomic risk is independent of age, we did not include age in any of the models considered.

We categorized the genetic susceptibility to cardiometabolic diseases using polygenic scores (PGSs). To define the genomic risk for lipid abnormalities, blood pressure abnormalities, and obesity, we used the PGS Catalog [49] to identify relevant polygenic risk scores corresponding to the 3 targets. Specifically, we identified 14 PGS for lipid abnormalities (PGS000060, PGS000061, PGS000062, PGS000063, PGS000065, PGS000115, PGS000192, PGS000309, PGS000310, PGS000311, PGS000340, PGS000677, PGS000688, and PGS000699), 2 for blood pressure abnormalities (PGS000301 and PGS000302), and 1 for obesity (PGS000298). Additional details of the selection process are provided in Section SI-3 (Multimedia Appendix 1).

For each of the 3 targets, we labeled a participant as having high genomic risk if their scores for any of the relevant PGS were in the top or bottom decile (refer to Section SI-3, Multimedia Appendix 1 for how the direction of a PGS is determined), which we term as the 90/10 cut-off. For instance, the high genomic risk group for lipid abnormalities would include members with high-risk scores for at least 1 of the 14 lipid-related PGS. The modeling of these targets and statistical comparison of the performance of different model types were identical to the earlier process described for the clinical risk targets.

To evaluate the sensitivity of the chosen percentile cut-offs for genomic risk scores, we repeated the above analyses for 2 additional sets of cut-offs, namely the 80/20 and 85/15 cut-offs.

### Illustrative Profiling Based on Clinical Events

Finally, we examined the connections between high-resolution wearable-derived features and actualized cardiometabolic disease events for participants not in our training set of 321. Among these participants, we considered those who actualized cardiometabolic disease events indicated by a primary diagnosis of cardiovascular disease, dyslipidemia, and hypertension (as per International Classification of Diseases, 10^th^ Revision codes listed in Table S3 in Multimedia Appendix 1). As this set of events spans a broad range of cardiometabolic conditions, anyRISKoutof9 is the closest surrogate marker. Hence, we chose to focus our profiling on the wearable-derived feature set that was most strongly associated with anyRISKoutof9.

For participants selected per the abovementioned criteria, we examined demographic information, physical measurements, genomic risk of disease, and clinical risk markers alongside the wearable-derived features. To interpret how the different wearable-derived features contribute to the model predictions at the individual participant level, we computed the Shapley values (Φ) [50] of each feature using the *iml* package [51] in R and selected the 5 features with the highest absolute magnitude of Φ for each participant. We illustrate the profiles of the participants, the predictions made by the best-performing model for anyRISKoutof9, and the features that contribute most to these predictions for each selected participant.

### Software and Code Availability

All statistical analyses and modeling were performed using R Statistical Software (version 4.0.3; R Core Team 2020). Computation of resting heart rate was performed using R, but all other feature engineering efforts such as annotation of wearable time series recordings and derivation of summary features, as well as the generation of high-resolution features, were performed using Python (version 3.8.6).

All Python and R codes used in feature generation are available in Multimedia Appendix 2.

## Results

### Characteristics of High-Resolution Heart Rate Features From Wearables

**Figure 3:**
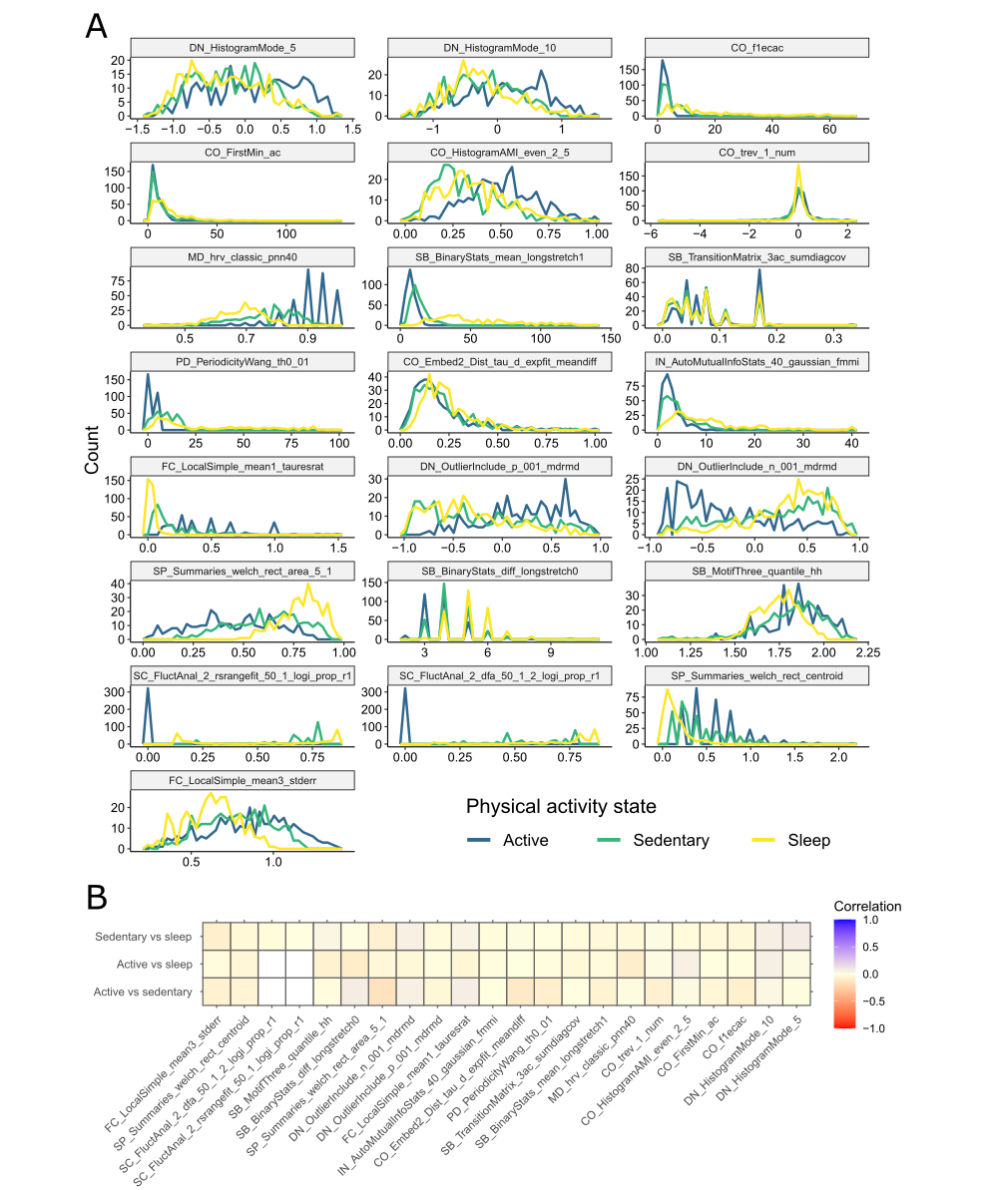
High-resolution (Canonical Time-series Characteristics 22 [Catch22]) wearable features from 3 different activity states. (A) Frequency polygons of the feature values based on the training set. The colors indicate activity states. (B) Pearson correlation coefficients between pairs of Catch22 features from different physical activity states (sleep, active, and sedentary). Two features from the active period (SC_FluctAnal_2_rsrangefit_50_1_logi_prop_r1 and SC_FluctAnal_2_dfa_50_1_2_logi_prop_r1) are uniformly 0; hence, correlation coefficients involving these 2 features are undefined (white squares).

Unlike summary statistics such as resting heart rate, which averages heart rate measurements across multiple days, our high-resolution feature sets provide more granularity on the heart rate time series dynamics during different physical activity states (sleep, active, and sedentary). Figure 3A illustrates the distributions of the high-resolution wearable feature values across the 321 participants (colored according to their respective activity states). Although the Catch22 algorithm was identically applied to each of the 3 activity segments, we observed that each feature exhibited distinct distributions across the 3 different activity states.

To study whether this difference holds at the participant level, we characterized the correlations among the high-resolution feature sets obtained during the 3 different activity states. For any given feature (eg, CO_trev1_num), we considered vectors of feature values for each physical activity state across the population (eg, CO_trev1_num.active, CO_trev1_num.sedentary, and CO_trev1_num.sleep). We then calculated the Pearson correlation between these feature vectors for each pair of the activity states. This analysis revealed that the feature values from the different activity states were poorly correlated (Figure 3B). In fact, the largest absolute correlation coefficient among any of the pairs was 0.15. Taken together, these findings indicate that heart rate dynamics from different activity states contain distinct information.

### Predictive Value of Wearable-Derived Features for Clinical Targets

Having gained some intuition about the information contained within the wearable-derived feature sets, we considered their predictive value for the clinical markers of cardiometabolic disease risk. Specifically, we trained random forest models to use the different wearable-derived feature sets to classify each of the 4 cardiometabolic disease risk targets. We performed comparative analyses to evaluate the predictive value of the different wearable-derived feature sets for classification of the 4 cardiometabolic disease risk targets.

First, we compared the OOB performance of the models trained using different feature sets for each clinical risk marker target (Table 4). For each target, the best-performing model was based on one of the high-resolution wearable feature sets (HighRes.ActiveSeg, HighRes.SedenSeg, or HighRes.SleepSeg). Specifically, for anyRISKoutof9, the HighRes.SedenSeg model was the best-performing model, with 17.9% and 7.36% lower Brier scores than baselines based on age and gender and resting heart rate, respectively (*P*<.001 in each case). This finding highlights the predictive value of high-resolution information within wearable heart rate time series recordings.

Second, we observed that heart rate dynamics extracted from different activity level segments have differential predictive potential for the various targets, as evidenced by the statistically significant differences between Brier scores (*P*<.001) of the HighRes.ActiveSeg, HighRes.SedenSeg, and HighRes.SleepSeg models (Table 4). Of the 3 model types, HighRes.SedenSeg performs best for lipid abnormalities, obesity, and anyRISKoutof9, whereas HighRes.ActiveSeg performs best for blood pressure abnormalities.

**Figure 4:**
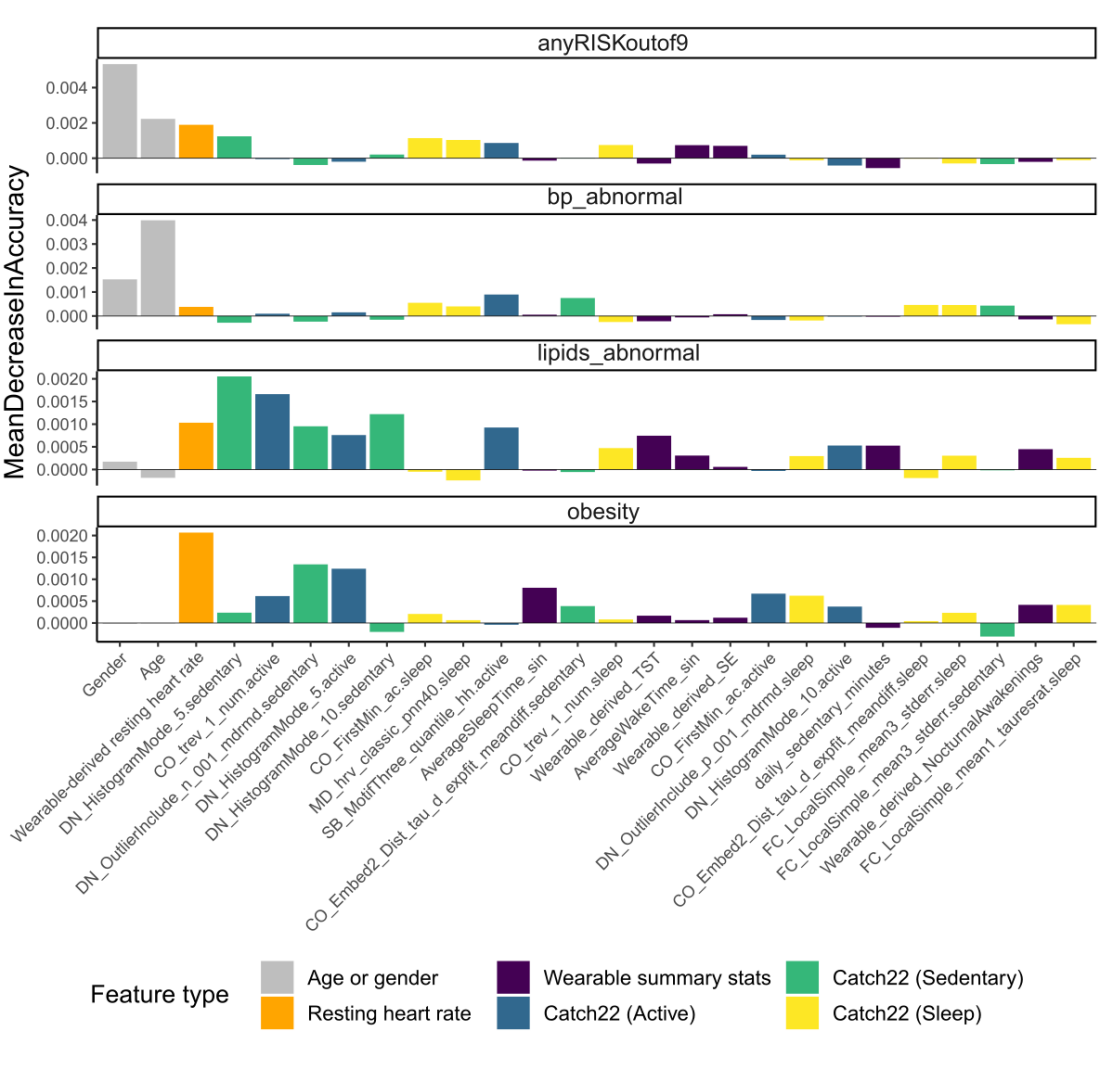
Random forest variable importance. The variable importance of each feature for prediction of the 4 cardiometabolic disease risk targets. We averaged each importance value across 200 simulations and used the results to rank the top 10 features to retain for each cardiometabolic disease risk target. This resulted in a total of 26 features across all 4 targets, as shown in the figure. Catch22: Canonical Time-series Characteristics 22.

Third, to comparatively evaluate contributions from individual wearable-derived features, we trained models that used all features available to predict each cardiometabolic disease risk target and ranked the variable importance in each case. Figure 4 shows the variable importance plots. It is clear that different features affect the performance of the models for each of the 4 targets. For instance, age and gender are the top 2 drivers of model performance for the anyRISKoutof9 target but are not among the top 10 features for both lipids_abnormal and obesity targets. Furthermore, we found that heart rate dynamics from different activity states contained distinct information on cardiometabolic disease risk. For example, the DN_HistogramMode_5 feature from the sedentary and active segments was important for predicting cardiometabolic disease risk markers but the DN_HistogramMode_5 feature from the sleep segment was not (Figure 4).

Fourth, we observed that the top 10 features for each of the 4 targets included features from all 6 feature types (age and gender, wearable-derived resting heart rate, wearable summary statistics, and the 3 sets of high-resolution features from Table 1). This suggests that risk prediction models using wearable-derived features may not exclusively rely on only one of the different feature sets or any one feature drawn from these feature sets. Rather, a collection of different wearable-derived high-resolution heart rate features from distinct activity states is essential for accurately predicting the multiplicity of cardiometabolic disease risk targets.

**Table 4:** Model performance on cardiometabolic risk targets. Out-of-bag model performance for each of the 5 model types computed for the 4 targets. A smaller Brier score indicates a better performing model for a given target.

	Baseline^a^, mean (SD)	RestingHR^b^, mean (SD)	HighRes.ActiveSeg^c^, mean (SD)	HighRes.SedenSeg^c^, mean (SD)	HighRes.SleepSeg^c^, mean (SD)	SummaryStats, mean (SD)
anyRISKoutof9	0.291 (−5.87×10^−4^)	0.258 (7.7×10^−4^)	0.253 (8.52×10^−4^)	0.239 (−9×10^−4^)	0.245 (8.43×10^−4^)	0.247 (7.66×10^−4^)
bp_abnormal	0.227 (4.79×10^−4^)	0.223 (5.61×10^−4^)	0.217 (7.88×10^−4^)	0.222 (8.14×10^−4^)	0.225 (8.32×10^−4^)	0.225 (7.9×10^−4^)
obesity	0.246 (6.64×10^−4^)	0.227 (7.91×10^−4^)	0.221 (8.92×10^−4^)	0.214 (9.34×10^−4^)	0.226 (8.64×10^−4^)	0.227 (8.54×10^−4^)
lipids_abnormal	0.271 (5.84×10^−4^)	0.261 (6.64×10^−4^)	0.238 (8.08×10^−4^)	0.225 (7.58×10^−4^)	0.241 (8.27×10^−4^)	0.236 (7.3×10^−4^)

^a^For each risk target, the Brier scores of the baseline model were significantly different from those of all other models (*P*<.001).

^b^For each risk target, Brier scores of the resting heart rate model (RestingHR) were significantly different from all other models (*P*<.001).

^c^For each risk target, Brier scores of the 3 HighRes models were significantly different from each other (*P*<.001).

### Associations Between Wearable-Derived Features and Genomic Risk Markers

To further interpret the information contained within the wearable-derived features, we sought to understand how they relate to the genetic predispositions for cardiometabolic diseases. Specifically, we examined the degree of information overlap between the different wearable-derived features (Table 1) and the genomic risk of cardiometabolic conditions. For each pairing between the different wearable-derived feature sets and the 3 genomic risk targets, we trained random forest models and used their Brier scores as indirect measures of the strength of the associations.

The results are presented in Table 5. For each of the 3 abnormality types, we observed that the high-resolution wearable features were more strongly associated with genomic risk levels than sex and resting heart rate (improvement of 11.9%-22.0% in Brier scores; *P*<.001). We highlight that the trends against baseline and resting heart rate were relatively insensitive to the polygenic risk score threshold used to define high versus low genomic risk (Section SI-4, Multimedia Appendix 1). These results suggest that, in comparison with standard measures, high-resolution features from wearables are better able to represent subtle physiological dynamics related to the genomic risk for cardiometabolic disease.

**Table 5:** Degree of association with genomic risk targets. Out-of-bag performance for each of the 5 model types computed for the 3 targets. A smaller Brier score indicates better performing model for a given target.

	Baseline^a^, mean (SD)	RestingHR^b^, mean (SD)	HighRes.ActiveSeg, mean (SD)	HighRes.SedenSeg, mean (SD)	HighRes.SleepSeg, mean (SD)	SummaryStats, mean (SD)
Blood pressure	0.248 (2.0×10^−3^)	0.245 (8.55×10^−4^)	0.215 (1.08×10^−3^)	0.214 (1.09×10^−3^)	0.215 (9.93×10^−4^)	0.212 (9.64×10^−4^)
Obesity	0.245 (2.31×10^−3^)	0.246 (9.03×10^−4^)	0.205 (1.15×10^−3^)	0.192 (1.06×10^−3^)	0.199 (1.21×10^−3^)	0.203 (1.06×10^−3^)
Lipids	0.294 (3.02×10^−3^)	0.308 (6.36×10^−4^)	0.254 (9.07×10^−4^)	0.254 (8.82×10^−4^)	0.259 (8.92×10^−4^)	0.268 (8.86×10^−4^)

^a^For each risk target, the Brier scores of the baseline model were significantly different from all other models (*P*<.001).

^b^For each risk target, Brier scores of the resting heart rate model (RestingHR) were significantly different from those of the 3 HighRes and SummaryStats models (*P*<.001).

### Illustrative Profiles of Participants With Cardiometabolic Events

Finally, we examined the relationship between the wearable-derived feature set most predictive for anyRISKoutof9 and actualized cardiometabolic events. We focused on participants not in our training set and filtered participants with data for the feature set most predictive for anyRISKoutof9 (ie, Catch22 [Sedentary] feature set, based on the abovementioned results). This yielded 197 candidate participants for illustrative profiling. Among these participants, only 5 participants actualized events with primary diagnoses for cardiometabolic conditions (as specified in Table S3 in Multimedia Appendix 1).

Table 6 provides demographic, genetic, and clinical risk profiles along with physical measurements and important wearable features for these 5 participants (A-E). All the participants were aged 54 to 61 years. Of the 5 participants, 4 (80%) were male. Only 1 (20%) participant was obese. We now present the findings on the predictive value of high-resolution wearable-derived features for these participants.

First, we describe participants with abnormalities in both genetic and clinical risk markers, namely participants A and B. Participant A had high genomic risk for all 3 conditions, presented abnormal values for most of the 9 clinical risk markers, and was also diagnosed with all 3 types of cardiometabolic conditions considered (cardiovascular disease, dyslipidemia, and hypertension). Participant B had a genomic risk for lipid and blood pressure abnormalities, abnormal lipid panel values, and a clinical diagnosis of dyslipidemia. While participant A had a wearable-derived resting heart rate slightly above the population average, participant B had a wearable-derived resting heart rate lower than the population average. However, in both cases, our HighRes.SedenSeg model predicted a positive anyRISKoutof9 outcome.

Second, we considered participants with no genomic risk but who presented with abnormal clinical risk markers, namely participant C. This participant had high blood pressure, abnormal cholesterol and blood glucose levels, a clinical diagnosis of dyslipidemia, and wearable-derived resting heart rate slightly above the population average value. However, we noted that our HighRes.SedenSeg model predicted a negative anyRISKoutof9 outcome. This could be due to modeling error or possibly be attributed to the absence of severe changes in heart rate dynamics given the normal genetic background and moderate wearable-derived resting heart rate value.

Third, we highlighted participants who did not exhibit any abnormalities in clinical risk markers and were borderline for cardiometabolic disease risk, namely participants D and E. Participant D only had a genomic risk for blood pressure. Participant E, on the other hand, appeared to have the most benign profile with low genomic risk for all 3 target conditions and normal values for all 9 clinical risk markers (with only the BMI being borderline high). Both participants had wearable-derived resting heart rate values that were lower than the population average. Although participants D and E had a seemingly low-risk profile by standard measures, they had clinical diagnoses of dyslipidemia and cardiovascular disease, respectively. Indeed, our HighRes.SedenSeg model predicted a positive anyRISKoutof9 outcome in each case.

Finally, inspecting the most important features (top 5 Shapley values) contributing to model predictions for anyRISKoutof9 in Table 6 reveals interesting patterns. While age and gender were (expectedly) consistent contributors to prediction scores for most participants, many Catch22 (Sedentary) features also contributed at comparable levels. For instance, DN_Histogram_Mode_5 was important for all 5 participants, whereas CO_Embed2_Dist_tau_d_expfit_meandiff and DN_OutlierInclude_p_001_mdrmd were important for 3 and 2 participants, respectively. In particular, DN_Histogram_Mode_5 was an important feature for most participants in this study. This feature takes on large values when the participant’s heart rate time series exhibits substantial deviations from the mean, which could occur when there are sustained or frequent oscillations with high amplitude. Although such deviations may be common in active states, their presence in sedentary states could forebode cardiovascular abnormalities, as was the case for these 5 participants. Beyond the consistent features noted above, there are other diverse high-resolution features among the top 5 most important contributors for different participants. This suggests that our high-resolution feature extraction approach offers a compact but sufficiently diverse set of predictive heart rate patterns, including those that are consistent across individual participants and those that can cater to participant-to-participant variability. Detailed Shapley Additive Explanations (SHAP) feature importance plots for each participant are provided in Section SI-5 in Multimedia Appendix 1.

**Table 6:** Illustrative profiles of 5 participants with actualized cardiometabolic events. Participant profiles include demographic information, type of cardiometabolic disease, key physical measurements, clinical and genomic risk markers, and the top 5 important wearable-derived heart rate features (as per Shapley values).

Participant profiles		Participant				
		A	B	C	D	E
**Demographics**						
	Age (years)	54	57	56	55	61
	Gender	Male	Male	Male	Female	Male
	Wearable-derived resting heart rate	72.8	58.2	73.0	69.0	55.7
**Clinical risk markers**						
	BMI (kg/m^2^)	28.05	18.79	21.27	22.95	25.95
	Blood pressure: SBP^a^/DBP^b^ (mm Hg)	166/109	108/65	164/105	112/48	133/89
	Glucose (mmol/L)	6.8	4.8	7.4	5.3	5.3
	Total cholesterol (mmol/L)	5.27	6.63	6.60	5.05	4.45
	anyRISKoutof9	True^c^	True	True	False^d^	False
**High genomic risk**						
	Lipids abnormalities	True	True	False	False	False
	Blood pressure abnormalities	True	True	False	True	False
	Obesity	True	False	False	False	False
**Actualized cardiometabolic events**						
	Cardiovascular disease	True	True	False	False	True
	Dyslipidemia	True	False	True	True	False
	Hypertension	True	False	False	False	False
**Important features for prediction**						
	CO_f1ecac.sedentary	False	False	False	True	False
	FC_LocalSimple_mean3_stderr.sedentary	True	False	False	False	False
	SB_MotifThree_quantile_hh.sedentary	True	False	False	False	False
	SB_TransitionMatrix_3ac_sumdiagcov.sedentary	False	False	False	True	False
	CO_trev_1_num.sedentary	False	False	False	False	True
	CO_HistogramAMI_even_2_5.sedentary	False	False	True	False	False
	DN_OutlierInclude_p_001_mdrmd.sedentary	True	True	False	False	False
	CO_Embed2_Dist_tau_d_expfit_meandiff.sedentary	False	True	False	True	True
	DN_HistogramMode_10.sedentary	False	False	True	False	False
	DN_HistogramMode_5.sedentary	True	True	True	True	True
	Gender	True	True	True	False	True
	Age (years)	False	True	True	True	True

^a^SBP: systolic blood pressure.

^b^DBP: diastolic blood pressure.

^c^True indicates true or that there is a presence of categorical variables.

^d^False indicates false or absence of categorical variables.

## Discussion

### Principal Findings

Consumer wearables enable the recording of rich high-resolution physiological dynamics in free-living conditions, but how these data relate to health and disease is not fully understood. We introduced a principled framework to derive high-resolution heart rate features from consumer wearable recordings, and applied our approach to a data set containing multidimensional cardiometabolic health parameters from healthy volunteers. Our results show that, in comparison with typical summary statistics, high-resolution features resolving temporal dynamics and activity-dependent patterns in heart rate have stronger associations with modifiable risk markers and inherent genetic predispositions for cardiometabolic disease alike. Our findings imply that these high-resolution digital phenotypes from consumer wearables can provide a more granular picture of cardiometabolic health and disease states, which could have potential use in cardiometabolic health screening and disease management.

Our framework addresses key challenges in mining wearable data recorded in free-living conditions. Unlike clean data from controlled experimental settings, real-world wearable recordings tend to be irregular, contain missing stretches [29], lack clean context annotations, and have variable lengths. As such, analyses based on the naive application of general purpose time series feature extraction methods [36,39,52] may not have ecological validity [53]. To address this gap and derive meaningful physiological dynamics from wearable time series recordings, our feature extraction framework standardizes handling of data irregularities and encodes contextual information about the underlying activity level and physiological state (Figures 1-3). This conceptual framework, although demonstrated here with the Catch22 method [39], is agnostic to the choice of the feature representation method for time series data [36,37]. Furthermore, in contrast to black-box feature learning methods based on large labeled data sets [31], our approach yields more interpretable time series features with smaller unlabeled data sets.

Our framework provides many possibilities for gaining new insights from wearable recordings. Our analyses, using multimodal wearable, genomic, and clinical data from healthy volunteers, highlight 2 possibilities.

First, our results revealed new relationships between high-resolution heart rate dynamics from wearables and the risk of cardiometabolic disease. Most previous studies correlated clinically obtained measures of heart rate dynamics, such as heart rate variability, exercise capacity, and heart rate recovery, with disease risk or outcomes [54-56]. In contrast, our results revealed that heart rate dynamics recorded by consumer wearables, when processed appropriately, are also predictive of cardiometabolic disease risk (Tables 4-6; Figure 4). Furthermore, we found that heart rate dynamics from different activity states contain distinct information about specific cardiometabolic conditions (Table 4; Figures 3 and 4). For example, heart rate patterns in sedentary states are more related to abnormalities in lipid levels and obesity, whereas those in active states may be more related to abnormalities in blood pressure readings (Table 4). These findings highlight the value addition of assessing physiology in free-living activity states (beyond controlled clinical settings) for disease risk monitoring and management [57].

Second, our study provides new perspectives on the interrelations between wearable recordings and genetic predispositions in cardiometabolic diseases. Although there has been a longstanding interest in probing gene-lifestyle interactions and their additive effects on cardiovascular disease [58-60], such studies have had limited visibility on physiology in free-living conditions. We found surprising connections (Table 5) between high-resolution wearable-derived feature sets and genetic predispositions for cardiometabolic disease. As these associations did not appear to depend on the presence or absence of manifest clinical risk markers, we posit that high-resolution phenotypes from wearables may capture subtle subclinical physiological changes stemming from latent predispositions to disease.

### Limitations

Although the uniquely multimodal nature of our data enables us to uncover many novel insights on high-resolution wearable phenotypes, limitations of data set size and cohort design present some challenges. First, it was infeasible to conduct full-scale gene-environment interaction studies [61-63]; or train state-of-the-art machine learning models with large feature sets. Second, as the risk of cardiometabolic disease is highly multifactorial, the limited visibility on relevant physical and lifestyle factors constrains the absolute predictive accuracy of all models presented. For instance, we had limited input on regular exercise habits as the observation span was less than a week, as well as limited overlap between key lifestyle indicators and wearable recordings (eg, only 9 participants who smoked had valid wearable records). Finally, as our study included only a small number of participants with actualized cardiometabolic events, we could not perform quantitative analyses to relate wearable phenotypes with clinical events. Future work based on larger cohorts [64] with more targeted study designs could address some of these limitations and enable cross-cohort validation of our current findings.

### Conclusions

In conclusion, we demonstrated that high-resolution digital phenotypes based on heart rate patterns in wearable recordings provide important insights into physiology in free-living conditions. Our results revealed that these measures are associated with both genetic and clinical risk markers of cardiometabolic disease and have additional predictive value beyond wearable-derived summary statistics and clinical measures of cardiometabolic health. Hence, our work expands possibilities to use digital phenotypes from consumer wearables as readily accessible indicators of cardiometabolic health and disease and motivates new approaches for quantitative scoring of cardiometabolic disease risk. Future studies could expand our findings to even higher resolution digital phenotypes that can be extracted from recordings with newer generations of wearable devices [65,66] and target evaluations for precision screening, health monitoring, and disease management applications.

## Appendix

Multimedia Appendix 1 Supplementary information.

Multimedia Appendix 2 Supplementary data: code for feature generation.

## Abbreviations

Catch22: Canonical Time-series Characteristics 22
MDA: mean decrease in accuracy
OOB: out-of-bag
PGS: polygenic score
SHAP: Shapley Additive Explanations

## References

[1] Vogels EA (2020). About one-in-five Americans use a smart watch or fitness tracker. Pew Research Center.

[2] Li X, Dunn J, Salins D, Zhou G, Zhou W, Schüssler-Fiorenza Rose SM, Perelman D, Colbert E, Runge R, Rego S, Sonecha R, Datta S, McLaughlin T, Snyder MP (2017). Digital health: tracking physiomes and activity using wearable biosensors reveals useful health-related information. PLoS Biol.

[3] Mishra T, Wang M, Metwally AA, Bogu GK, Brooks AW, Bahmani A, Alavi A, Celli A, Higgs E, Dagan-Rosenfeld O, Fay B, Kirkpatrick S, Kellogg R, Gibson M, Wang T, Hunting EM, Mamic P, Ganz AB, Rolnik B, Li X, Snyder MP (2020). Pre-symptomatic detection of COVID-19 from smartwatch data. Nat Biomed Eng.

[4] Lim WK, Davila S, Teo JX, Yang C, Pua CJ, Blöcker C, Lim JQ, Ching J, Yap JJ, Tan SY, Sahlén A, Chin CW, Teh BT, Rozen SG, Cook SA, Yeo KK, Tan P (2018). Beyond fitness tracking: the use of consumer-grade wearable data from normal volunteers in cardiovascular and lipidomics research. PLoS Biol.

[5] Quisel T, Foschini L, Kale DC (2016). Intra-day activity better predicts chronic conditions. Workshop on Machine Learning for Health.

[6] Bayoumy K, Gaber M, Elshafeey A, Mhaimeed O, Dineen EH, Marvel FA, Martin SS, Muse ED, Turakhia MP, Tarakji KG, Elshazly MB (2021). Smart wearable devices in cardiovascular care: where we are and how to move forward. Nat Rev Cardiol.

[7] Garcia-Ceja E, Riegler M, Nordgreen T, Jakobsen P, Oedegaard KJ, Tørresen J (2018). Mental health monitoring with multimodal sensing and machine learning: a survey. Pervasive Mob Comput.

[8] Cooney MT, Vartiainen E, Laatikainen T, Juolevi A, Dudina A, Graham IM (2010). Elevated resting heart rate is an independent risk factor for cardiovascular disease in healthy men and women. Am Heart J.

[9] Fox K, Borer JS, Camm AJ, Danchin N, Ferrari R, Lopez Sendon JL, Steg PG, Tardif JC, Tavazzi L, Tendera M, Heart Rate Working Group (2007). Resting heart rate in cardiovascular disease. J Am Coll Cardiol.

[10] Cook S, Togni M, Schaub MC, Wenaweser P, Hess OM (2006). High heart rate: a cardiovascular risk factor?. Eur Heart J.

[11] Rykov Y, Thach T, Dunleavy G, Roberts AC, Christopoulos G, Soh CK, Car J (2020). Activity tracker-based metrics as digital markers of cardiometabolic health in working adults: cross-sectional study. JMIR Mhealth Uhealth.

[12] Bumgarner JM, Lambert CT, Hussein AA, Cantillon DJ, Baranowski B, Wolski K, Lindsay BD, Wazni OM, Tarakji KG (2018). Smartwatch algorithm for automated detection of atrial fibrillation. J Am Coll Cardiol.

[13] Tison GH, Sanchez JM, Ballinger B, Singh A, Olgin JE, Pletcher MJ, Vittinghoff E, Lee ES, Fan SM, Gladstone RA, Mikell C, Sohoni N, Hsieh J, Marcus GM (2018). Passive detection of atrial fibrillation using a commercially available smartwatch. JAMA Cardiol.

[14] Turakhia MP, Hoang DD, Zimetbaum P, Miller JD, Froelicher VF, Kumar UN, Xu X, Yang F, Heidenreich PA (2013). Diagnostic utility of a novel leadless arrhythmia monitoring device. Am J Cardiol.

[15] Galloway CD, Valys AV, Petterson FL, Gundotra VP, Treiman DL, Albert DE, Dillon JJ, Attia ZI, Friedman PA (2018). Non-invasive detection of hyperkalemia with a smartphone electrocardiogram and artificial intelligence. J Am Coll Cardiol.

[16] Galloway CD, Valys AV, Shreibati JB, Treiman DL, Petterson FL, Gundotra VP, Albert DE, Attia ZI, Carter RE, Asirvatham SJ, Ackerman MJ, Noseworthy PA, Dillon JJ, Friedman PA (2019). Development and validation of a deep-learning model to screen for hyperkalemia from the electrocardiogram. JAMA Cardiol.

[17] Quer G, Gouda P, Galarnyk M, Topol EJ, Steinhubl SR (2020). Inter- and intraindividual variability in daily resting heart rate and its associations with age, sex, sleep, BMI, and time of year: retrospective, longitudinal cohort study of 92,457 adults. PLoS One.

[18] Sopic D, Aminifar A, Aminifar A, Atienza D (2018). Real-time event-driven classification technique for early detection and prevention of myocardial infarction on wearable systems. IEEE Trans Biomed Circuits Syst.

[19] Attia ZI, Kapa S, Lopez-Jimenez F, McKie PM, Ladewig DJ, Satam G, Pellikka PA, Enriquez-Sarano M, Noseworthy PA, Munger TM, Asirvatham SJ, Scott CG, Carter RE, Friedman PA (2019). Screening for cardiac contractile dysfunction using an artificial intelligence-enabled electrocardiogram. Nat Med.

[20] Strath SJ, Rowley TW (2018). Wearables for promoting physical activity. Clin Chem.

[21] Teo JX, Davila S, Yang C, Hii AA, Pua CJ, Yap J, Tan SY, Sahlén A, Chin CW, Teh BT, Rozen SG, Cook SA, Yeo KK, Tan P, Lim WK (2019). Digital phenotyping by consumer wearables identifies sleep-associated markers of cardiovascular disease risk and biological aging. Commun Biol.

[22] Guo C, Lu M, Chen J (2020). An evaluation of time series summary statistics as features for clinical prediction tasks. BMC Med Inform Decis Mak.

[23] Schroeder EB, Liao D, Chambless LE, Prineas RJ, Evans GW, Heiss G (2003). Hypertension, blood pressure, and heart rate variability: the Atherosclerosis Risk in Communities (ARIC) study. Hypertension.

[24] Liao D, Carnethon M, Evans GW, Cascio WE, Heiss G (2002). Lower heart rate variability is associated with the development of coronary heart disease in individuals with diabetes: the atherosclerosis risk in communities (ARIC) study. Diabetes.

[25] Kotecha D, New G, Flather MD, Eccleston D, Krum H (2011). 61 Five-min heart rate variability can predict obstructive angiographic coronary disease. Heart.

[26] Harvard Health Publishing Staff (2021). Heart rate variability: how it might indicate well-being. Harvard Health.

[27] Oura Team (2020). Heart Rate During Sleep: Look for These 3 Patterns. Oura.

[28] Pevnick JM, Birkeland K, Zimmer R, Elad Y, Kedan I (2018). Wearable technology for cardiology: an update and framework for the future. Trends Cardiovasc Med.

[29] Nelson BW, Allen NB (2019). Accuracy of consumer wearable heart rate measurement during an ecologically valid 24-hour period: intraindividual validation study. JMIR Mhealth Uhealth.

[30] Godino JG, Wing D, de Zambotti M, Baker FC, Bagot K, Inkelis S, Pautz C, Higgins M, Nichols J, Brumback T, Chevance G, Colrain IM, Patrick K, Tapert SF (2020). Performance of a commercial multi-sensor wearable (Fitbit Charge HR) in measuring physical activity and sleep in healthy children. PLoS One.

[31] Ballinger B, Hsieh J, Singh A, Sohoni N, Wang J, Tison GH, Marcus GM, Sanchez JM, Maguire C, Olgin JE, Pletcher MM (2018). DeepHeart: semi-supervised sequence learning for cardiovascular risk prediction. Proc AAAI Conf Artif Intell.

[32] Tison GH, Singh AC, Ohashi DA, Hsieh JT, Ballinger BM, Olgin JE, Marcus GM, Pletcher MJ (2017). Abstract 21042: cardiovascular risk stratification using off-the-shelf wearables and a multi-task deep learning algorithm. Circulation.

[33] National Heart Centre Singapore (2016). Effects of physical activity, ambulatory blood pressure and calcium score on cardiovascular health in normal people (SingHEART) – Report No.: NCT02791152. Clinical Trials.

[34] Yap J, Lim WK, Sahlén A, Chin CW, Chew KM, Davila S, Allen J, Goh V, Tan SY, Tan P, Lam CS, Cook SA, Yeo KK (2019). Harnessing technology and molecular analysis to understand the development of cardiovascular diseases in Asia: a prospective cohort study (SingHEART). BMC Cardiovasc Disord.

[35] Fulcher BD, Jones NS (2014). Highly comparative feature-based time-series classification. IEEE Trans Knowl Data Eng.

[36] Fulcher BD, Jones NS (2017). hctsa: a computational framework for automated time-series phenotyping using massive feature extraction. Cell Syst.

[37] Christ M, Braun N, Neuffer J, Kempa-Liehr AW (2018). Time series FeatuRe extraction on basis of scalable hypothesis tests (tsfresh – a Python package). Neurocomputing.

[38] Christ M, Kempa-Liehr AW, Feindt M (2017). Distributed and parallel time series feature extraction for industrial big data applications. arXiv.

[39] Lubba CH, Sethi SS, Knaute P, Schultz SR, Fulcher BD, Jones NS (2019). catch22: CAnonical Time-series CHaracteristics. Data Min Knowl Disc.

[40] Bent B, Goldstein BA, Kibbe WA, Dunn JP (2020). Investigating sources of inaccuracy in wearable optical heart rate sensors. NPJ Digit Med.

[41] Lex A, Gehlenborg N, Strobelt H, Vuillemot R, Pfister H (2014). UpSet: visualization of intersecting sets. IEEE Trans Vis Comput Graph.

[42] Conway JR, Lex A, Gehlenborg N (2017). UpSetR: an R package for the visualization of intersecting sets and their properties. Bioinformatics.

[43] Breiman L (2001). Random forests. Mach Learn.

[44] Liaw A, Wiener M (2002). Classification and regression by RandomForest. R News.

[45] Chen C, Liaw A, Breiman L (2004). Using Random Forest to Learn Imbalanced Data. Department of Statistics, University of California, Berkeley.

[46] Brier GW (1950). Verification of forecasts expressed in terms of probability. Mon Wea Rev.

[47] Breiman L (1996). Out-of-bag estimation. University of California Berkeley.

[48] Benjamini Y, Hochberg Y (1995). Controlling the false discovery rate: a practical and powerful approach to multiple testing. J R Stat Soc Ser B Methodol.

[49] The Polygenic Score (PGS) Catalog. PGS Catalog.

[50] Lundberg S, Lee SI (2016). An unexpected unity among methods for interpreting model predictions. Advances in Neural Information Processing Systems.

[51] Molnar C, Casalicchio G, Bischl B (2018). iml: an R package for interpretable machine learning. J Open Source Softw.

[52] Pouromran F, Radhakrishnan S, Kamarthi S (2021). Exploration of physiological sensors, features, and machine learning models for pain intensity estimation. PLoS One.

[53] Hu B, Chen Y, Keogh E (2015). Classification of streaming time series under more realistic assumptions. Data Min Knowl Disc.

[54] Kubota Y, Chen LY, Whitsel EA, Folsom AR (2017). Heart rate variability and lifetime risk of cardiovascular disease: the Atherosclerosis Risk in Communities Study. Ann Epidemiol.

[55] van de Vegte YJ, van der Harst P, Verweij N (2018). Heart rate recovery 10 seconds after cessation of exercise predicts death. J Am Heart Assoc.

[56] Georgiopoulou VV, Kalogeropoulos AP, Chowdhury R, Binongo JN, Bibbins-Domingo K, Rodondi N, Simonsick EM, Harris T, Newman AB, Kritchevsky SB, Butler J, Health ABC Study (2017). Exercise capacity, heart failure risk, and mortality in older adults: the health ABC study. Am J Prev Med.

[57] Niemelä M, Kangas M, Farrahi V, Kiviniemi A, Leinonen AM, Ahola R, Puukka K, Auvinen J, Korpelainen R, Jämsä T (2019). Intensity and temporal patterns of physical activity and cardiovascular disease risk in midlife. Prev Med.

[58] Khera AV, Emdin CA, Drake I, Natarajan P, Bick AG, Cook NR, Chasman DI, Baber U, Mehran R, Rader DJ, Fuster V, Boerwinkle E, Melander O, Orho-Melander M, Ridker PM, Kathiresan S (2016). Genetic risk, adherence to a healthy lifestyle, and coronary disease. N Engl J Med.

[59] Said MA, Verweij N, van der Harst P (2018). Associations of combined genetic and lifestyle risks with incident cardiovascular disease and diabetes in the UK Biobank study. JAMA Cardiol.

[60] (2018). Individual access to genomic disease risk factors has a beneficial impact on lifestyles. ScienceDaily.

[61] Ottman R (1996). Gene-environment interaction: definitions and study designs. Prev Med.

[62] Thomas D (2010). Methods for investigating gene-environment interactions in candidate pathway and genome-wide association studies. Annu Rev Public Health.

[63] Li J, Li X, Zhang S, Snyder M (2019). Gene-environment interaction in the era of precision medicine. Cell.

[64] Sudlow C, Gallacher J, Allen N, Beral V, Burton P, Danesh J, Downey P, Elliott P, Green J, Landray M, Liu B, Matthews P, Ong G, Pell J, Silman A, Young A, Sprosen T, Peakman T, Collins R (2015). UK biobank: an open access resource for identifying the causes of a wide range of complex diseases of middle and old age. PLoS Med.

[65] Muggeridge DJ, Hickson K, Davies AV, Giggins OM, Megson IL, Gorely T, Crabtree DR (2021). Measurement of heart rate using the polar OH1 and Fitbit charge 3 wearable devices in healthy adults during light, moderate, vigorous, and sprint-based exercise: validation study. JMIR Mhealth Uhealth.

[66] Lubitz SA, Faranesh AZ, Atlas SJ, McManus DD, Singer DE, Pagoto S, Pantelopoulos A, Foulkes AS (2021). Rationale and design of a large population study to validate software for the assessment of atrial fibrillation from data acquired by a consumer tracker or smartwatch: the Fitbit heart study. Am Heart J.

[67] National Supercomputing Centre.

