# Supplementary material for: High-Resolution Digital Phenotypes From Consumer Wearables and Their Applications in Machine Learning of Cardiometabolic Risk Markers: Cohort Study
Source: J Med Internet Res. 2022 Jul 29;24(7):e34669. doi: 10.2196/34669 (PMC9377462; doi:10.2196/34669)
Supplement: Multimedia Appendix 1 [file jmir_v24i7e34669_app1.pdf]

High-Resolution Digital Phenotypes From Consumer Wearables and Their Applications in Machine Learning of Cardiometabolic Risk Markers: Cohort Study

Weizhuang Zhou*[,1], Yu En Chan*[,1], Chuan Sheng Foo[1], Jingxian Zhang[1], Jing Xian Teo[2], Sonia Davila[2,3,4], Weiting Huang[5], Jonathan Yap[5,6], Stuart Cook[4], Patrick Tan[2,7,8,9], Calvin Woon-Loong Chin[5,6], Khung Keong Yeo[2,5,6], Weng Khong Lim[+,2,3,7], Pavitra Krishnaswamy[+,1]

*Denotes equal contributions
[+]Correspondence: wengkhong.lim@duke-nus.edu.sg; pavitrak@i2r.a-star.edu.sg

Author Affiliations
[1] Institute for Infocomm Research, Agency for Science Technology and Research (A*STAR), Singapore
[2] SingHealth Duke-NUS Institute of Precision Medicine, Singapore
[3] SingHealth Duke-NUS Genomic Medicine Centre, Singapore
[4] Cardiovascular and Metabolic Disorders Program, Duke-NUS Medical School, Singapore, Singapore
[5] Department of Cardiology, National Heart Centre Singapore, Singapore
[6] Duke-NUS Medical School, Singapore
[7] Cancer and Stem Biology Program, Duke-NUS Medical School, Singapore
[8] Cancer Science Institute of Singapore, National University of Singapore, Singapore
[9] Genome Institute of Singapore, Agency for Science Technology and Research (A*STAR), Singapore

Supplementary Information

Table of Contents

Table S1: Description of Catch22 Features ... 3

Table S2: Wearable Data Summary Statistics ... 5

Table S3: Description of ICD codes for Illustrative Profiling ... 6

SI-1: Determination of Time Series Segment Lengths for Catch22 Features ... 7

- Summary Statistics ... 8
  - Summary stats of longest continuous time series in active period ... 8
  - Summary stats of longest continuous time series in sedentary period ... 8
  - Summary stats of longest continuous time series in sleep period ... 8
- Average Coefficient of Variation (CV) ... 8
  - Heatmap of Average CV in active period ... 9
  - Heatmap of Average CV in sedentary period ... 10
  - Heatmap of Average CV in sleep period ... 11

SI-2: Distribution of Cardiometabolic Risk Targets ... 12

SI-3: Selection and Processing of Polygenic Risk Scores ... 13

- Selected PGS and Mapped Trait Ontology ... 13
  - Lipids Abnormality ... 13
  - Blood Pressure Abnormality ... 13
  - Obesity ... 13
- Annotation of High or Low Risk Score ... 13

SI-4: Sensitivity Analysis - Association between Wearable Features and Genomic Risk Markers ... 14

- Number of Subjects for Genomic Risk Targets ... 14
- Brier Scores of Different Model Types ... 15
  - 80/20 Cut-offs ... 15
  - 85/15 Cut-offs ... 15

SI-5: SHAP variable importance plots for Subjects A-E ... 16

Table S1: Description of Catch22 Features

NB: The feature descriptions in this table is reproduced from Table 1 of Lubba *et al.* (*catch22*: CAnonical Time-series CHaracteristics. *Data Min Knowl Disc* **33**, 1821–1852 (2019)), under the terms of the Creative Commons Attribution 4.0 International License (http://creativecommons.org/licenses/by/4.0/).

ID	Feature Name	Description	Feature Category
1	DN_HistogramMode_5	Mode of z-scored distribution (5-bin histogram)	Distribution
2	DN_HistogramMode_10	Mode of z-scored distribution (10-bin histogram)	
3	DN_OutlierInclude_p_001_mdrmd	Time intervals between successive extreme events above the mean	Extreme events
4	DN_OutlierInclude_n_001_mdrmd	Time intervals between successive extreme events below the mean	
5	SB_BinaryStats_mean_longstretch1	Longest period of consecutive values above the mean	Symbolic
6	SB_BinaryStats_diff_longstretch0	Longest period of successive incremental decreases	
7	SB_MotifThree_quantile_hh	Shannon entropy of two successive letters in equiprobable 3-letter symbolization	
8	SB_TransitionMatrix_3ac_sumdiagcov	Trace of covariance of transition matrix between symbols in 3-letter alphabet	
9	CO_f1ecac	First 1/e crossing of autocorrelation function	Linear autocorrelation and periodicity
10	CO_FirstMin_ac	First minimum of autocorrelation function	
11	SP_Summaries_welch_rect_area_5_1	Total power in lowest fifth of frequencies in the Fourier power spectrum	
12	SP_Summaries_welch_rect_centroid	Centroid of the Fourier power spectrum	

13	FC_LocalSimple_mean3_stderr	Mean error from a rolling 3-sample mean forecasting	
14	PD_PeriodicityWang_th0_01	Periodicity measure	
15	CO_trev_1_num	Time-reversibility statistic, $\langle (x_{t+1}-x_t)^3 \rangle_t$	Nonlinear autocorrelation
16	CO_HistogramAMI_even_2_5	Automutual information, $m=2,\tau=5$	
17	IN_AutoMutualInfoStats_40_gaussian_fmmi	First minimum of the automutual information function	
18	MD_hrv_classic_pnn40	Proportion of successive differences exceeding 0.04σ	Successive differences
19	FC_LocalSimple_mean1_tauresrat	Change in correlation length after iterative differencing	
20	CO_Embed2_Dist_tau_d_expfit_meandiff	Exponential fit to successive distances in 2D embedding space	
21	SC_FluctAnal_2_dfa_50_1_2_logi_prop_r1	Proportion of slower timescale fluctuations that scale with DFA (50% sampling)	Fluctuation analysis
22	SC_FluctAnal_2_rsrangefit_50_1_logi_prop_r1	Proportion of slower timescale fluctuations that scale with linearly rescaled range fits	

For visualisations of the features, please see: https://github.com/chlubba/catch22/wiki/Feature-Descriptions

Table S2: Wearable Data Summary Statistics

Feature Set Type	Features	Description
Summary Statistics for Mean Daily Physical Activity Durations	Wearable_derived_TST	Average wearable-derived total sleep time
	daily_sedentary_minutes	Average minutes/day spent in sedentary period
	daily_active_minutes	Average minutes/day spent in active period
Summary Statistics from Device Logs	Wearable_derived_Nocturnal Awakenings_minutes	Average daily minutes of nocturnal awakenings
	Wearable_derived_Nocturnal Awakenings	Average number of nocturnal awakenings
	Wearable_derived_SE	Wearable-derived sleep efficiency score (from Fitbit)
Average Wake and Sleep Times	AverageWakeTime_sin	Mean waking time, sine transformed
	AverageWakeTime_cos	Mean waking time, cosine transformed
	AverageSleepTime_sin	Mean bedtime, sine transformed
	AverageSleepTime_cos	Mean bedtime, cosine transformed

Sleep efficiency used to be a score that could be retrieved by the Fitbit API. The formula has never been published by Fitbit, although a comparison of actual sleep records against the retrieved scores indicates that it is defined as:

$$\text{Sleep Efficiency} = \frac{minutesAsleep}{minutesAsleep + minutesAwake} \times 100$$

Table S3: Description of ICD codes for Illustrative Profiling

Cardiovascular Disease

	ICD 10-Code	Description
1	I200	Unstable angina
2	I208	Other forms of angina pectoris
3	I211	Acute transmural myocardial infarction of inferior wall
4	I214	Acute subendocardial myocardial infarction
5	I2510	Atherosclerotic heart disease of native coronary artery without angina pectoris
6	I2511	Atherosclerotic heart disease, of native coronary artery
7	I255	Ischemic cardiomyopathy
8	I258	Other forms of chronic ischaemic heart disease
9	I259	Chronic ischaemic heart disease, unspecified
10	I420	Dilated cardiomyopathy
11	I440	Atrioventricular block, first degree
12	I447	Left bundle-branch block, unspecified
13	I451	Other and unspecified right bundle-branch block
14	I458	Other specified conduction disorders
15	I471	Supraventricular tachycardia
16	I48	Atrial fibrillation and flutter
17	I493	Ventricular premature depolarisation
18	I495	Sick sinus syndrome
19	I498	Other specified cardiac arrhythmias
20	R000	Tachycardia, unspecified
21	R001	Bradycardia, unspecified

Dyslipidemia

	ICD 10-Code	Description
1	E780	Pure hypercholesterolemia
2	E781	Pure hyperglyceridemia
3	E782	Mixed hyperlipidemia
4	E783	Hyperchylomicronemia
5	E784	Other hyperlipidemia
6	E785	Hyperlipidemia, unspecified
7	E786	Lipoprotein deficiency

Hypertension

	ICD-10 Code	Description
1	I10	Essential (primary) hypertension
2	I11	Hypertensive heart disease
3	I12	Hypertensive chronic kidney disease
4	I13	Hypertensive heart and chronic kidney disease

Obesity

	ICD-10 Code	Description
1	E668	Other obesity
2	E669	Obesity, unspecified

SI-1: Determination of Time Series Segment Lengths for Catch22 Features

To generate the annotations of the activity levels for a subject, we considered only days with at least 20 hours of valid step count/heart rate measurements per day. Only 642 out of the theoretical maximum of 692 subjects fulfilled this requirement. For each of the 642 subjects, we obtained the longest continuous heart rate time series in active, sedentary, and sleep periods. The median lengths of the time series are 31mins, 1h 45mins, and 7h 45mins respectively as shown in the tables below. To determine what the ideal time length should be for generating catch22 features in each of those three periods, we ran the following series of experiments. First, we computed catch22 on sliding windows of varying lengths for each of the three activity periods (active: [10 min, 15 min, 20 min, 25 min, 30 min], sedentary: [10 min, 20 min, 30 min, 1 h], sleep: [10 min, 20 min, 30 min, 1h, 3h, 5h]). We then calculated the coefficient of variation (CV) of each feature for each individual, and averaged it across all eligible individuals. Finally, we picked the window length that gives the most stable results (i.e. least number of extreme CV values) for each activity state. 20 min, 1h and 5h gave the most stable results for active, sedentary and sleep state respectively as shown in the heat maps. Thus, we used the first 20 min, 1h, and 5h of the longest continuous heart rate time series in active, sedentary and sleep periods respectively, to generate three sets of catch22 features for each subject.

Summary Statistics

Summary stats of longest continuous time series in active period

Count	642
Mean	0 days 00:38:00.654205607
Std	0 days 00:22:26.685840823
Min	0 days 00:07:00
25%	0 days 00:22:00
50%	0 days 00:31:00
75%	0 days 00:47:00
Max	0 days 02:39:00

Summary stats of longest continuous time series in sedentary period

Count	629
Mean	0 days 03:07:24.133545310
Std	0 days 02:48:02.781473861
Min	0 days 00:16:00
25%	0 days 01:10:00
50%	0 days 01:45:00
75%	0 days 04:38:00
Max	0 days 19:37:00

Summary stats of longest continuous time series in sleep period

Count	598
Mean	0 days 07:07:49.966555183
Std	0 days 01:45:29.474139290
Min	0 days 00:06:00
25%	0 days 06:14:30
50%	0 days 07:24:00
75%	0 days 08:10:45
Max	0 days 13:48:00

Average Coefficient of Variation (CV)

The CV was computed using the following formula:

$$CV = \frac{\text{Standard Deviation}}{\text{Mean}}$$

For some window sizes, some of the feature values were uniformly zero. This led to undefined CV, which are represented by grey boxes.

Heatmap of Average CV in active period

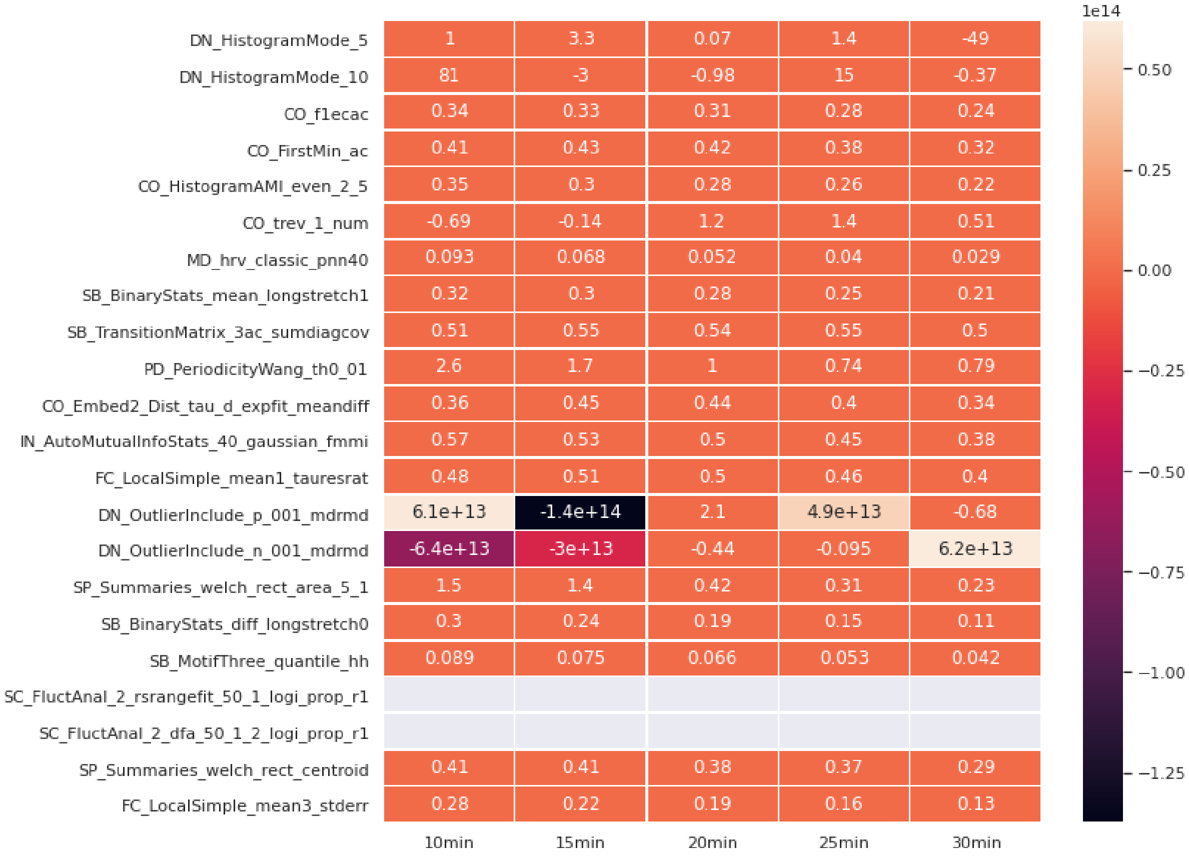

The 20-minute window length do not have extreme mean CV like the other lengths in the active period, hence it is the most stable.

Heatmap of Average CV in sedentary period

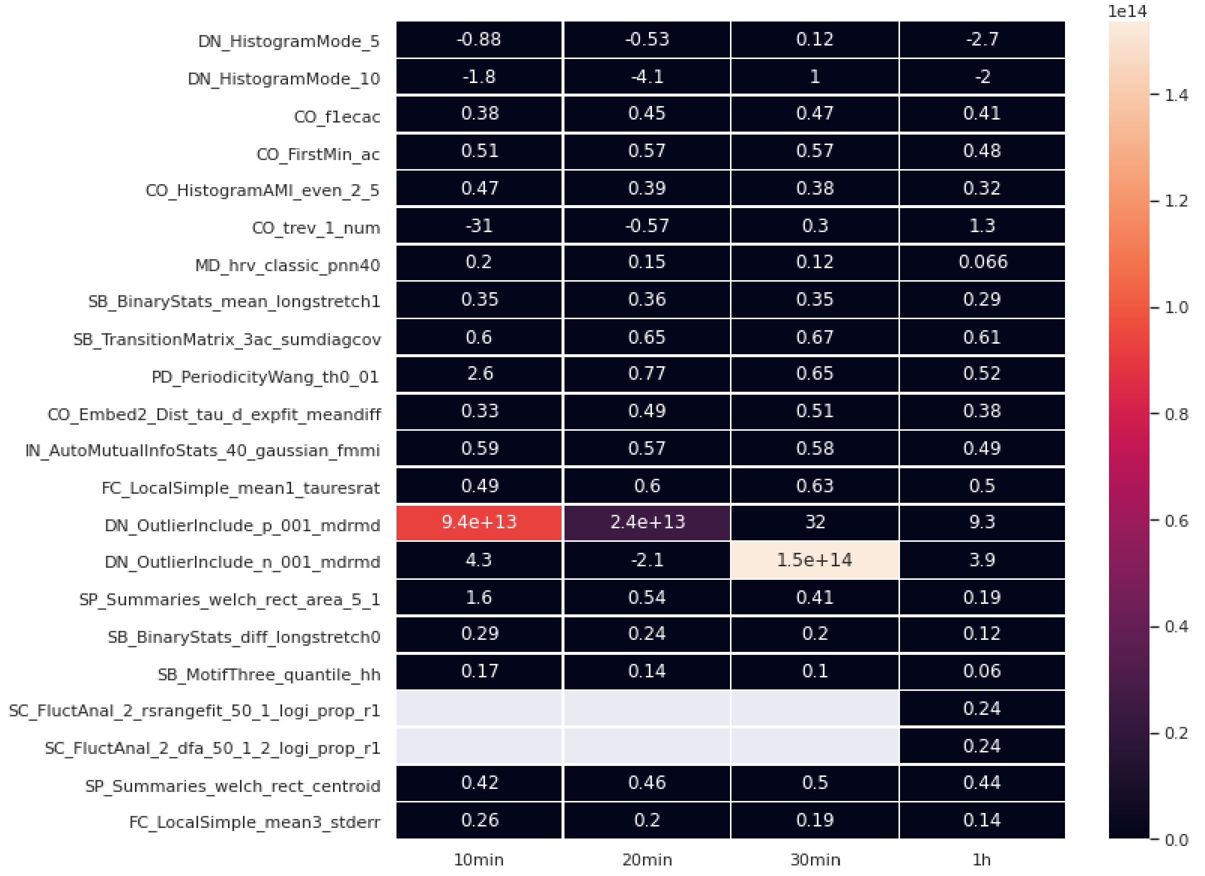

The 1-hour window length do not have extreme mean CV like the other lengths in the sedentary period, hence it is the most stable.

Heatmap of Average CV in sleep period

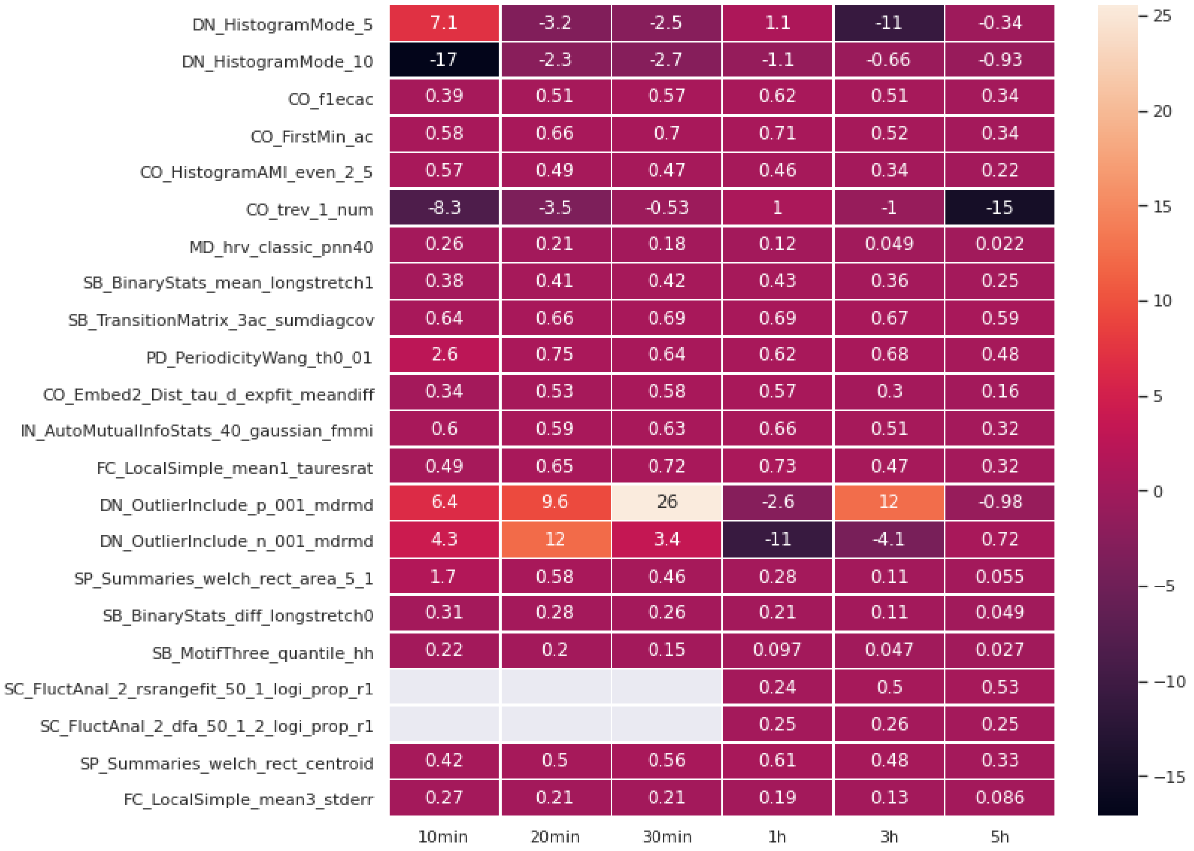

The 5-hour window length only has one feature that has an extreme mean CV in the sleep period, hence it is the most stable out of all the window sizes.

SI-2: Distribution of Cardiometabolic Risk Targets

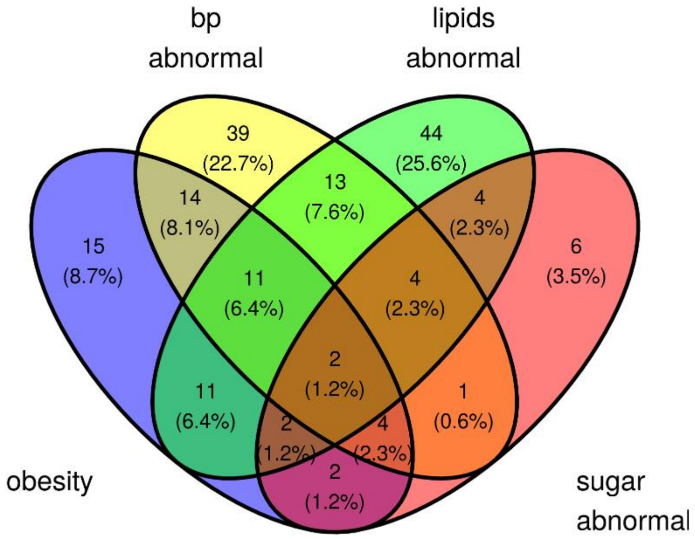

The training set for cardiometabolic risk targets consisted of 321 subjects. Of these, 172 subjects have at least one of the four major classes of abnormalities (obesity, blood pressure abnormalities, lipids abnormalities and sugar abnormalities). The above Venn diagram shows how this 172 subject group is distributed across different possible subsets of the four abnormality classes.

Due to the extremely small number of subjects in the "sugar abnormal" class, we did not train any models for this class. However, the subjects of the "sugar abnormal" class are included in the higher-order class "anyRISKoutof9".

SI-3: Selection and Processing of Polygenic Risk Scores

Polygenic risk scores with less than 20,000 variants from the PGS Catalog [50] were filtered based on the mapped trait ontology [51,52]. Eligible PGS were then validated against the PRISM cohort: we first determined the "direction" of a PGS by comparing the proportion of true cases (based on the laboratory measurements) amongst the subjects with scores below the 5th percentile and those with scores above the 95th percentile. Only PGS whose ratio of proportions was >=1.5 were retained.

Selected PGS and Mapped Trait Ontology

Lipids Abnormality

	PGS ID	Mapped Trait Ontology	Num. of Variants
1	PGS000060	high density lipoprotein cholesterol measurement	46
2	PGS000061	low density lipoprotein cholesterol measurement	37
3	PGS000062	total cholesterol measurement	52
4	PGS000063	triglyceride measurement	32
5	PGS000065	low density lipoprotein cholesterol measurement	103
6	PGS000115	low density lipoprotein cholesterol measurement	223
7	PGS000192	high density lipoprotein cholesterol measurement	9
8	PGS000309	high density lipoprotein cholesterol measurement	247
9	PGS000310	low density lipoprotein cholesterol measurement	194
10	PGS000311	total cholesterol measurement	234
11	PGS000340	low density lipoprotein cholesterol measurement	28
12	PGS000677	total cholesterol measurement	17,204
13	PGS000688	low density lipoprotein cholesterol measurement	16,184
14	PGS000699	triglyceride measurement	16,003

Blood Pressure Abnormality

	PGS ID	Mapped Trait Ontology	Num. of Variants
1	PGS000301	systolic blood pressure	970
2	PGS000302	diastolic blood pressure	962

Obesity

	PGS ID	Mapped Trait Ontology	Num. of Variants
1	PGS000298	Body mass index	941

Annotation of High or Low Risk Score

For PGS that are in the positive direction (i.e. larger scores means high proportion of true cases for abnormalities in the mapped trait), we considered subjects that have scores higher than the 90th percentile (top decile) as having high risk score. Conversely, for PGS that are in the negative direction, we considered subjects with scores smaller than the 10th percentile (bottom decile) as being high risk score. We assigned subjects to high and low risk groups for each PGS based on the above.

SI-4: Sensitivity Analysis - Association between Wearable Features and Genomic Risk Markers

The PGS risk groups in Table 5 of the main paper were defined by using the 90th (or 10th) percentile of the associated PGS as cut-offs. In order to determine if the obtained results were sensitive to these cut-off settings, we consider two other cut-offs and present the two subsections below.

Number of Subjects for Genomic Risk Targets

80/20 Cut-offs

Genomic Risk Targets	Number of Subjects with High Genomic Risk	Number of Subjects with Normal Genomic Risk
Lipids Abnormalities	238	83
Blood Pressure Abnormalities	79	242
Obesity	69	252

85/15 Cut-offs

Genomic Risk Targets	Number of Subjects with High Genomic Risk	Number of Subjects with Normal Genomic Risk
Lipids Abnormalities	220	101
Blood Pressure Abnormalities	67	254
Obesity	45	276

90/10 Cut-offs

Genomic Risk Targets	Number of Subjects with High Genomic Risk	Number of Subjects with Normal Genomic Risk
Lipids Abnormalities	169	152
Blood Pressure Abnormalities	40	281
Obesity	33	288

Brier Scores of Different Model Types

80/20 Cut-offs

	Baseline	*RHR*	*HighRes. ActiveSeg*	*HighRes. SedenSeg*	*HighRes. SleepSeg*	*SummaryStats*
Blood pressure	0.263 (2.27×10^{-3})	0.268 (6.02×10^{-4})	**0.229 (9.14×10^{-4})**	0.234 (7.97×10^{-4})	0.233 (9.02×10^{-4})	0.23 (8.50×10^{-4})
Obesity	0.229 (2.28×10^{-3})	0.253 (8.61×10^{-4})	0.212 (1.00×10^{-3})	**0.208 (9.76×10^{-4})**	0.211 (1.00×10^{-3})	0.213 (1.01×10^{-3})
Lipids	0.274 (1.68×10^{-3})	0.253 (5.60×10^{-4})	0.248 (8.77×10^{-4})	0.247 (8.14×10^{-4})	0.244 (7.75×10^{-4})	**0.243 (8.16×10^{-4})**

85/15 Cut-offs

	Baseline	*RHR*	*HighRes. ActiveSeg*	*HighRes. SedenSeg*	*HighRes. SleepSeg*	*SummaryStats*
Blood pressure	0.263 (1.83×10^{-3})	0.28 (6.98×10^{-4})	**0.237 (7.95×10^{-4})**	0.239 (8.48×10^{-4})	0.241 (9.36×10^{-4})	0.241 (8.08×10^{-4})
Obesity	0.265 (2.20×10^{-3})	0.253 (6.84×10^{-4})	0.231 (9.03×10^{-4})	**0.218 (8.32×10^{-4})**	0.222 (8.10×10^{-4})	0.222 (7.95×10^{-4})
Lipids	0.332 (4.02×10^{-3})	0.261 (6.18×10^{-4})	0.239 (8.25×10^{-4})	0.234 (8.03×10^{-4})	**0.232 (7.51×10^{-4})**	0.243 (9.42×10^{-4})

SI-5: SHAP variable importance plots for Subjects A-E

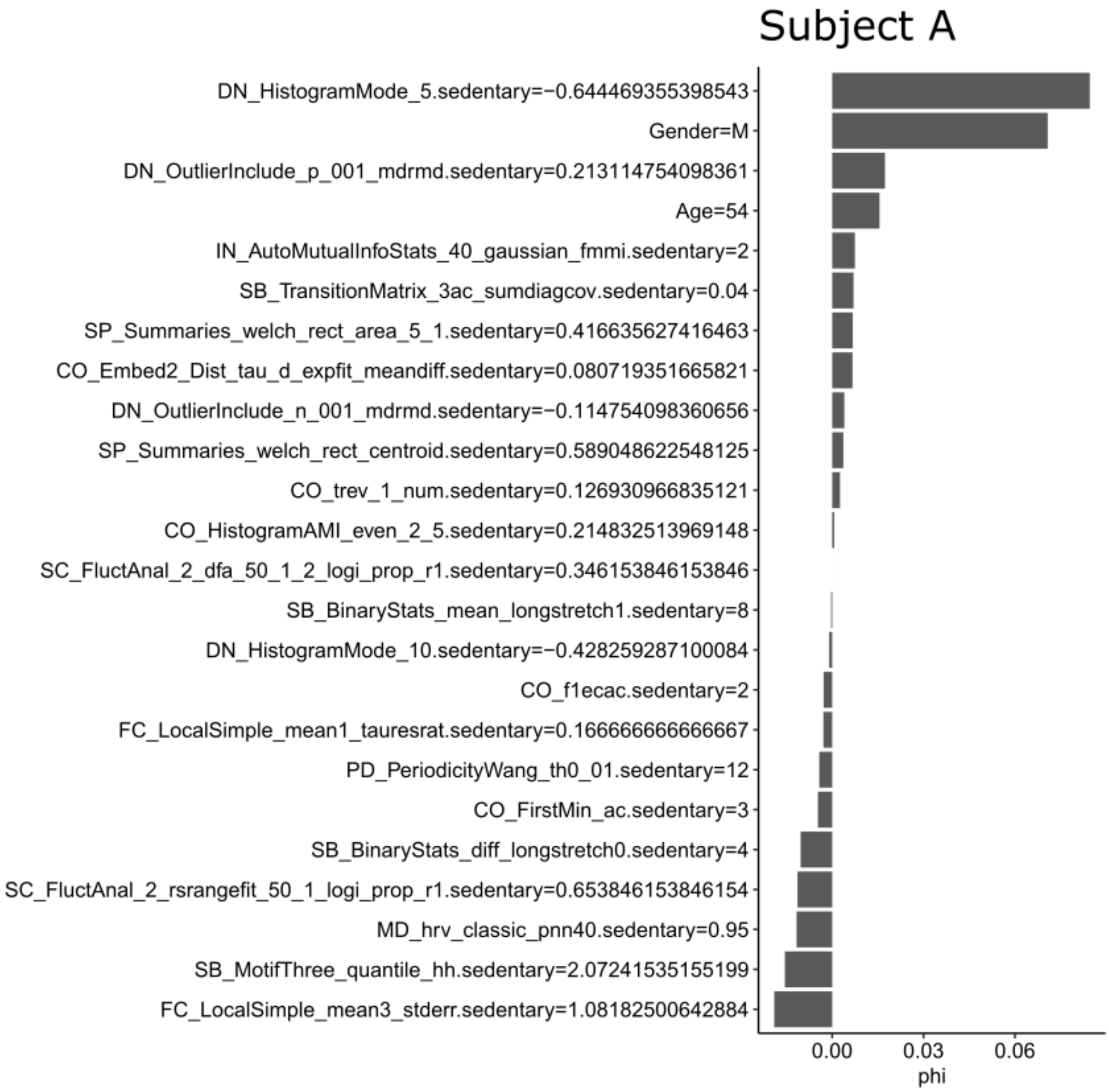

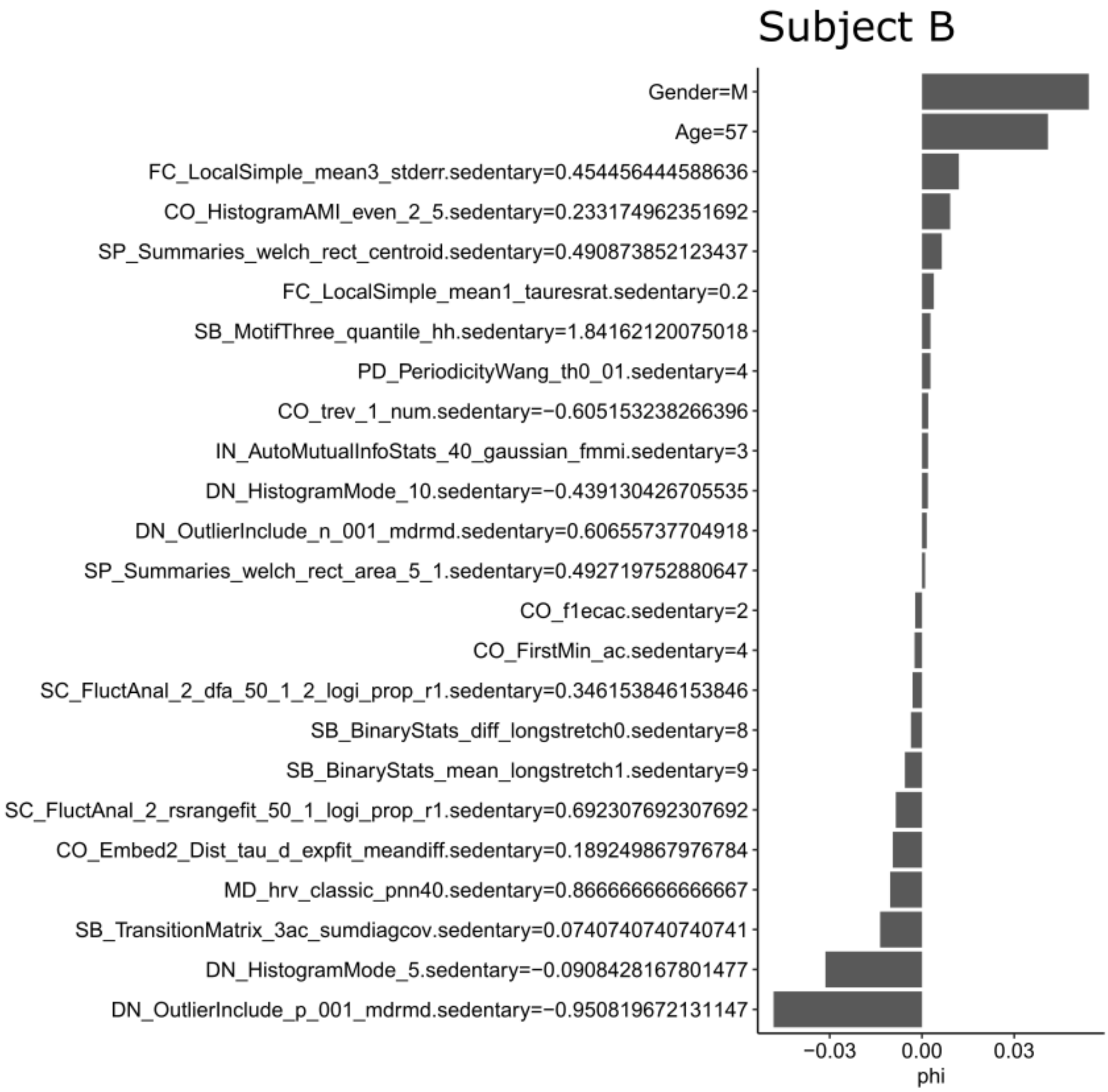

Subject B
Gender=M
Age=57
FC_LocalSimple_mean3_stderr.sedentary=0.454456444588636
CO_HistogramAMI_even_2_5.sedentary=0.233174962351692
SP_Summaries_welch_rect_centroid.sedentary=0.490873852123437
FC_LocalSimple_mean1_tauresrat.sedentary=0.2
SB_MotifThree_quantile_hh.sedentary=1.84162120075018
PD_PeriodicityWang_th0_01.sedentary=4
CO_trev_1_num.sedentary=−0.605153238266396
IN_AutoMutualInfoStats_40_gaussian_fmmi.sedentary=3
DN_HistogramMode_10.sedentary=−0.439130426705535
DN_OutlierInclude_n_001_mdrmd.sedentary=0.60655737704918
SP_Summaries_welch_rect_area_5_1.sedentary=0.492719752880647
CO_f1ecac.sedentary=2
CO_FirstMin_ac.sedentary=4
SC_FluctAnal_2_dfa_50_1_2_logi_prop_r1.sedentary=0.346153846153846
SB_BinaryStats_diff_longstretch0.sedentary=8
SB_BinaryStats_mean_longstretch1.sedentary=9
SC_FluctAnal_2_rsrangefit_50_1_logi_prop_r1.sedentary=0.692307692307692
CO_Embed2_Dist_tau_d_expfit_meandiff.sedentary=0.189249867976784
MD_hrv_classic_pnn40.sedentary=0.866666666666667
SB_TransitionMatrix_3ac_sumdiagcov.sedentary=0.0740740740740741
DN_HistogramMode_5.sedentary=−0.0908428167801477
DN_OutlierInclude_p_001_mdrmd.sedentary=−0.950819672131147
−0.03
0.00
0.03
phi

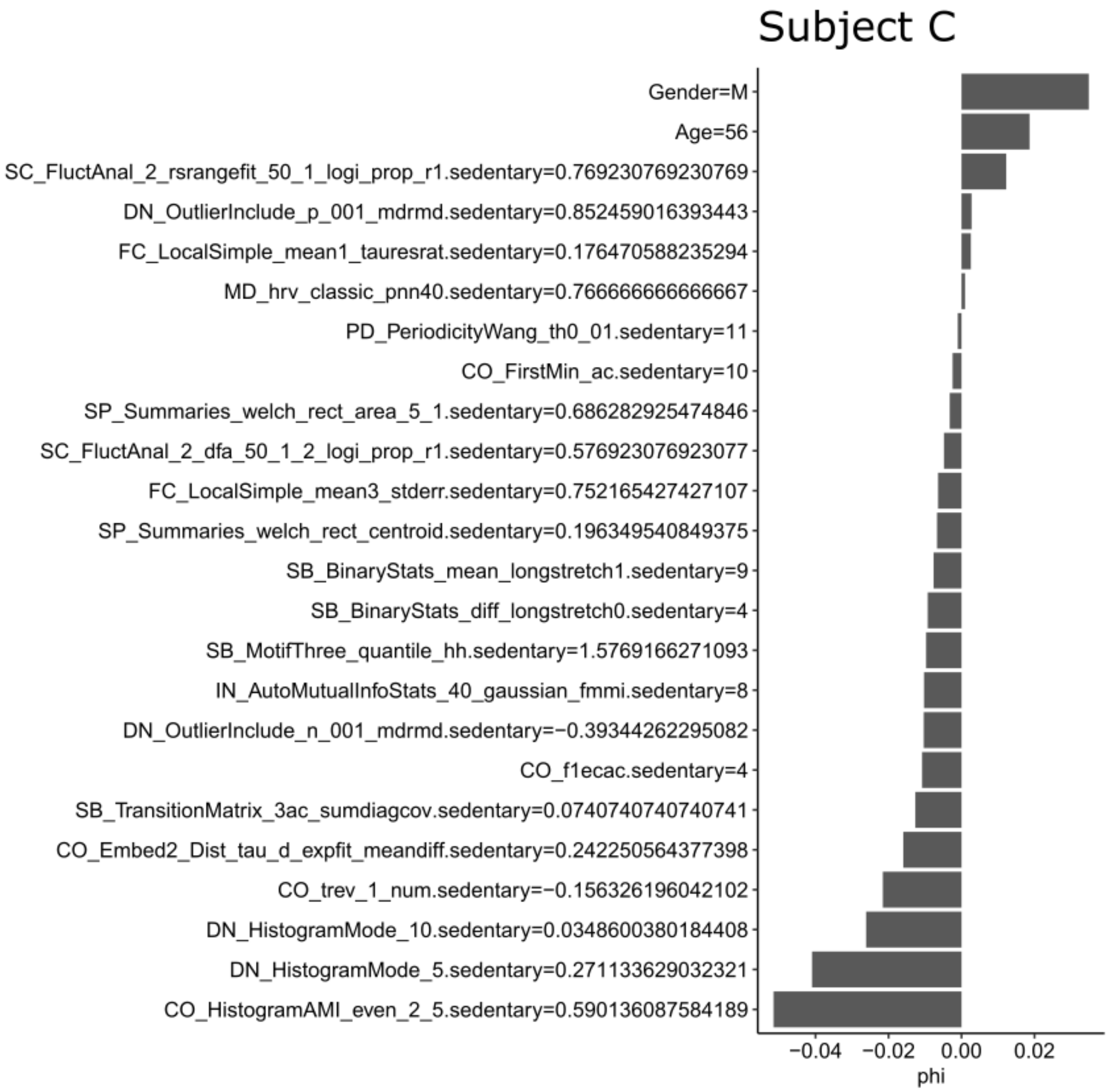
Subject C
Gender=M
Age=56
SC_FluctAnal_2_rsrangefit_50_1_logi_prop_r1.sedentary=0.769230769230769
DN_OutlierInclude_p_001_mdrmd.sedentary=0.852459016393443
FC_LocalSimple_mean1_tauresrat.sedentary=0.176470588235294
MD_hrv_classic_pnn40.sedentary=0.766666666666667
PD_PeriodicityWang_th0_01.sedentary=11
CO_FirstMin_ac.sedentary=10
SP_Summaries_welch_rect_area_5_1.sedentary=0.686282925474846
SC_FluctAnal_2_dfa_50_1_2_logi_prop_r1.sedentary=0.576923076923077
FC_LocalSimple_mean3_stderr.sedentary=0.752165427427107
SP_Summaries_welch_rect_centroid.sedentary=0.196349540849375
SB_BinaryStats_mean_longstretch1.sedentary=9
SB_BinaryStats_diff_longstretch0.sedentary=4
SB_MotifThree_quantile_hh.sedentary=1.5769166271093
IN_AutoMutualInfoStats_40_gaussian_fmmi.sedentary=8
DN_OutlierInclude_n_001_mdrmd.sedentary=−0.39344262295082
CO_f1ecac.sedentary=4
SB_TransitionMatrix_3ac_sumdiagcov.sedentary=0.0740740740740741
CO_Embed2_Dist_tau_d_expfit_meandiff.sedentary=0.242250564377398
CO_trev_1_num.sedentary=−0.156326196042102
DN_HistogramMode_10.sedentary=0.0348600380184408
DN_HistogramMode_5.sedentary=0.271133629032321
CO_HistogramAMI_even_2_5.sedentary=0.590136087584189
−0.04
−0.02
0.00
0.02
phi

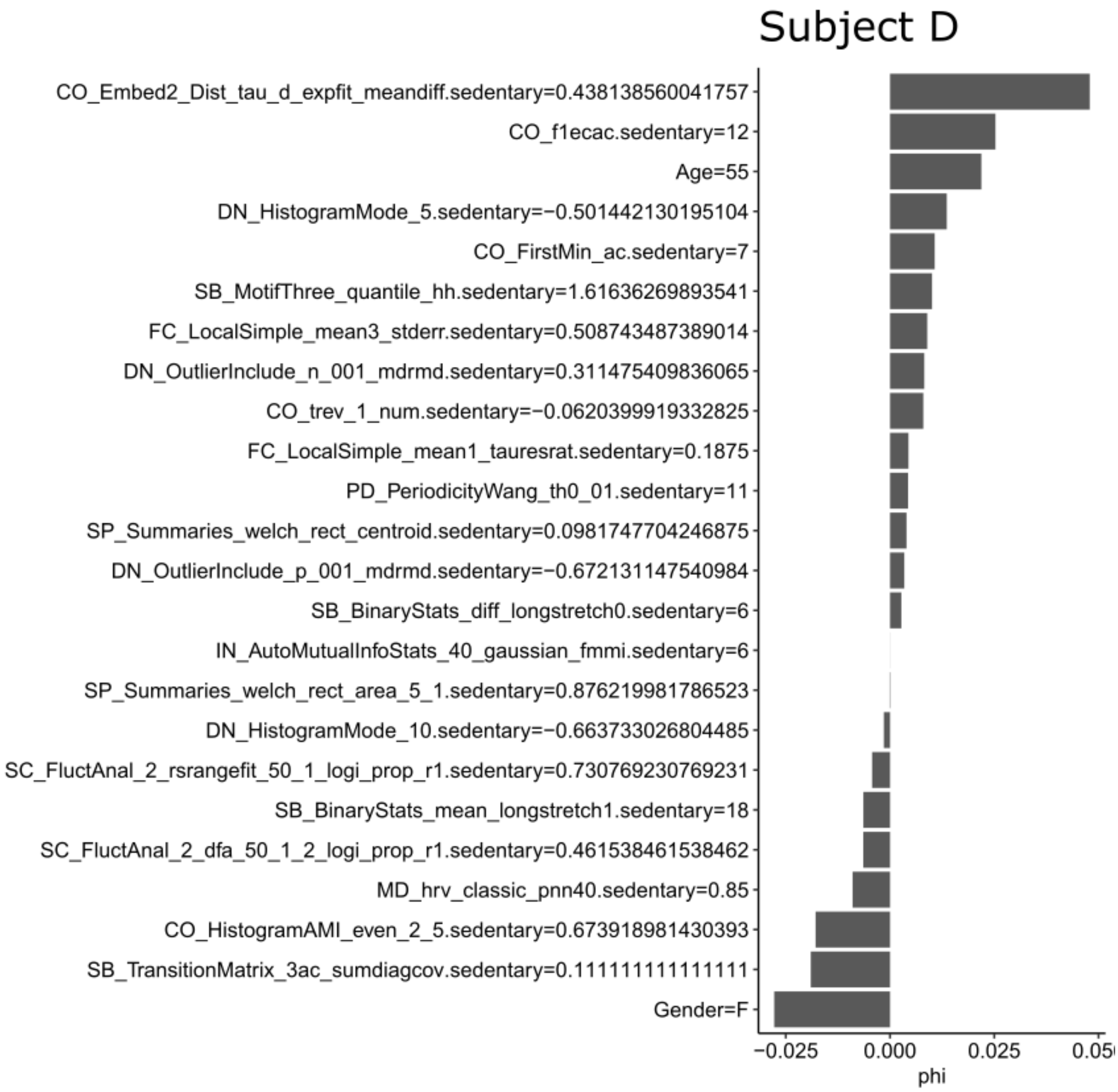

Subject D
CO_Embed2_Dist_tau_d_expfit_meandiff.sedentary=0.438138560041757
CO_f1ecac.sedentary=12
Age=55
DN_HistogramMode_5.sedentary=−0.501442130195104
CO_FirstMin_ac.sedentary=7
SB_MotifThree_quantile_hh.sedentary=1.61636269893541
FC_LocalSimple_mean3_stderr.sedentary=0.508743487389014
DN_OutlierInclude_n_001_mdrmd.sedentary=0.311475409836065
CO_trev_1_num.sedentary=−0.0620399919332825
FC_LocalSimple_mean1_tauresrat.sedentary=0.1875
PD_PeriodicityWang_th0_01.sedentary=11
SP_Summaries_welch_rect_centroid.sedentary=0.0981747704246875
DN_OutlierInclude_p_001_mdrmd.sedentary=−0.672131147540984
SB_BinaryStats_diff_longstretch0.sedentary=6
IN_AutoMutualInfoStats_40_gaussian_fmmi.sedentary=6
SP_Summaries_welch_rect_area_5_1.sedentary=0.876219981786523
DN_HistogramMode_10.sedentary=−0.663733026804485
SC_FluctAnal_2_rsrangefit_50_1_logi_prop_r1.sedentary=0.730769230769231
SB_BinaryStats_mean_longstretch1.sedentary=18
SC_FluctAnal_2_dfa_50_1_2_logi_prop_r1.sedentary=0.461538461538462
MD_hrv_classic_pnn40.sedentary=0.85
CO_HistogramAMI_even_2_5.sedentary=0.673918981430393
SB_TransitionMatrix_3ac_sumdiagcov.sedentary=0.111111111111111
Gender=F
−0.025
0.000
0.025
phi

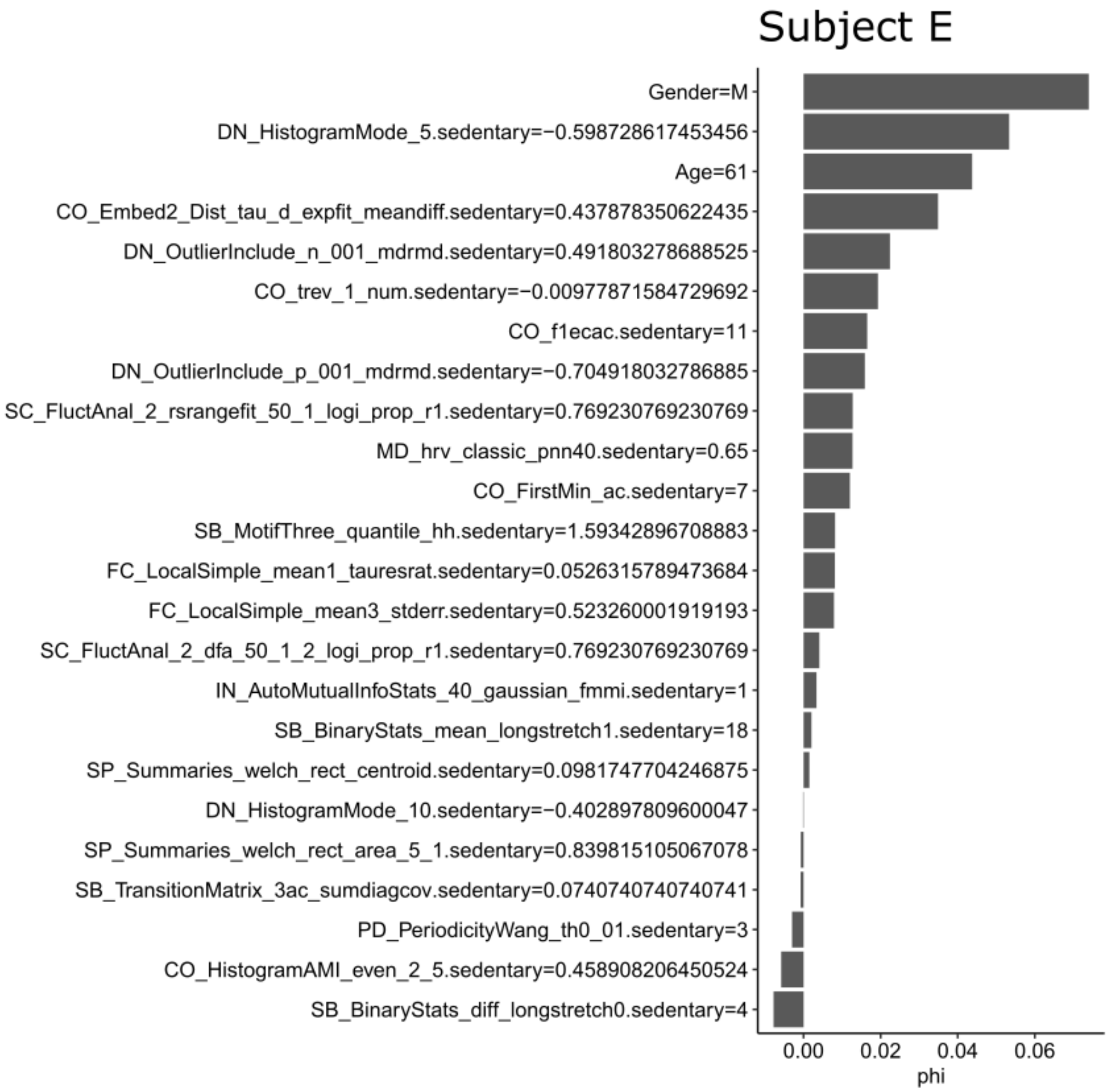
Subject E
Gender=M
DN_HistogramMode_5.sedentary=−0.598728617453456
Age=61
CO_Embed2_Dist_tau_d_expfit_meandiff.sedentary=0.437878350622435
DN_OutlierInclude_n_001_mdrmd.sedentary=0.491803278688525
CO_trev_1_num.sedentary=−0.00977871584729692
CO_f1ecac.sedentary=11
DN_OutlierInclude_p_001_mdrmd.sedentary=−0.704918032786885
SC_FluctAnal_2_rsrangefit_50_1_logi_prop_r1.sedentary=0.769230769230769
MD_hrv_classic_pnn40.sedentary=0.65
CO_FirstMin_ac.sedentary=7
SB_MotifThree_quantile_hh.sedentary=1.59342896708883
FC_LocalSimple_mean1_tauresrat.sedentary=0.0526315789473684
FC_LocalSimple_mean3_stderr.sedentary=0.523260001919193
SC_FluctAnal_2_dfa_50_1_2_logi_prop_r1.sedentary=0.769230769230769
IN_AutoMutualInfoStats_40_gaussian_fmmi.sedentary=1
SB_BinaryStats_mean_longstretch1.sedentary=18
SP_Summaries_welch_rect_centroid.sedentary=0.0981747704246875
DN_HistogramMode_10.sedentary=−0.402897809600047
SP_Summaries_welch_rect_area_5_1.sedentary=0.839815105067078
SB_TransitionMatrix_3ac_sumdiagcov.sedentary=0.0740740740740741
PD_PeriodicityWang_th0_01.sedentary=3
CO_HistogramAMI_even_2_5.sedentary=0.458908206450524
SB_BinaryStats_diff_longstretch0.sedentary=4
0.00
0.02
0.04
0.06
phi
